# Sex differences in cancer incidence persist across race and ethnicity

**DOI:** 10.1186/s13293-026-00902-z

**Published:** 2026-04-28

**Authors:** Julia J. Francis, Hormuzd A. Katki, Barry I. Graubard, Christopher A. Haiman, Loïc Le Marchand, Veronica Wendy Setiawan, Lynne R Wilkens, Anil Chaturvedi, Sarah S. Jackson

**Affiliations:** 1 Division of Cancer Epidemiology and Genetics, National Cancer Institute, 9609 Medical Center Drive, Rockville, 20850 MD USA; 2 Department of Population and Public Health Sciences, Keck School of Medicine, University of Southern California, Los Angeles, CA USA; 3 Center for Genetic Epidemiology, Keck School of Medicine, University of Southern California, Los Angeles, CA USA; 4 Epidemiology Program, University of Hawaii Cancer Center, Honolulu, HI USA

## Abstract

**Background:**

Males have a higher incidence of cancers at shared anatomic sites than females. We investigated whether sex differences in cancer incidence differed across race/ethnicity.

**Methods:**

Within the Multiethnic Cohort Study, we examined the association between sex and cancer incidence at 13 shared anatomic sites using Cox proportional hazards regression to estimate male-to-female hazard ratios (MF HRs) adjusting for cancer specific risk factors by race/ethnic group: African American, Japanese American, Native Hawaiian, Latino, and White. Statistical differences in the MF HRs by race/ethnicity for each site were assessed with an interaction term. The percentage of sex difference in cancer incidence that was statistically explained by the risk factors within race/ethnicity were quantified using a Peters-Belson approach.

**Results:**

Of the 178,036 participants, 46% were male and 54% were female. Adjusting for multiple risk factors did not eliminate the MF differences for any of the cancers examined. We found significant differences in MF HRs across race/ethnicity for cancers of the esophagus (Native Hawaiian MF HR 17.67, 95% CI 2.21–141.20; African American 1.28 0.69–2.36; *P* = 0.018), liver (Japanese American 1.28, 0.96–1.70; White 2.79, 1.81–4.30, *P* = 0.005) and thyroid (Latino 0.21, 0.13–0.35; White 0.83, 0.50–1.37, *P* = 0.005). Cancer risk factors explained 82% of the overall sex difference observed for lung cancer but 0% of the difference for gallbladder cancer.

**Discussion:**

Most non-sex-specific cancers have male predominance across race/ethnicity, which are not fully explained by cancer risk factors, suggesting there may be intrinsic sex differences driving these differences in cancer incidence.

**Supplementary Information:**

The online version contains supplementary material available at 10.1186/s13293-026-00902-z.

## Abstract (plain language summary)

Non-reproductive cancers are more common among males than females. Often this difference is attributed women’s healthier lifestyle because men are more likely to smoke and drink than women. However, previous research has found that cancer is still more common among men even when accounting for these differences in health behavior. These previous studies focused mostly on non-Hispanic White populations, so they did not look at cancer sex differences across race and ethnicity. There may be different sociocultural practices or external exposures that could not be accounted for in majority White populations. Therefore, we examined the sex difference in cancer risk in the Multiethnic Cohort Study accounting for differences in age, smoking, alcohol use, diet, family history, and chronic conditions. We looked at sex differences in cancer risk by five race and ethnic groups: non-Hispanic White, non-Hispanic Black, Hispanic, Japanese American, and Native Hawaiian. We found that these sex differences remained across racial and ethnic groups for 10 of the 13 cancers. These results suggest that the sex difference in cancer incidence many not be related to differences in lifestyle between men and women. These findings indicate there are biological mechanisms (like hormones, chromosomes, and immune response) that differ between the sexes that can cause cancer.

**Supplementary Information:**

The online version contains supplementary material available at 10.1186/s13293-026-00902-z.

## Highlights

- Males have 2–3 times the cancer incidence of females for most non-reproductive cancers.
- We examined the male-to-female incidence rate ratio for 13 cancer sites across five race and ethnic groups in the US adjusting for age, smoking, alcohol use, diet, family history, and chronic conditions.
- We found significant sex differences in cancer incidence remained across race and ethnicity for 10 of 13 cancer sites examined.
- Cancer risk factors statistically explained 0%-82% of the overall sex difference in incidence depending on the cancer site.
- That sex differences persisted across race and ethnicity for most cancer sites implicates sex-specific biological mechanisms, rather than external factors, in cancer incidence differences between males and females.

**Supplementary Information:**

The online version contains supplementary material available at 10.1186/s13293-026-00902-z.

## Introduction

The National Cancer Institute estimates that 40.5% of individuals living in the United States will be diagnosed with cancer at some point in their lifetime with annual incidence rates per 100,000 of 500 among males and 437 among females [1, 2]. At shared anatomic sites, males are 2–3 times more likely a develop cancer than females, after adjustment for known risk factors, with the exception of meningioma, thyroid and gallbladder cancers, which have a female predominance [3–5]. However, the higher site-specific cancer incidence in males compared to females remains unexplained.

The immune system plays a crucial role in cancer development. The effects of aging and fluctuating sex hormone levels across life stages are known intrinsic factors linked to sex differences in immune responses [6]. There are also external determinants of health such as environmental exposures to carcinogens, oncogenic infectious agents, socio-cultural factors, and healthcare utilization among other lifestyle factors that may influence cancer development. Additionally, many of these risk factors have differential prevalence among men and women. However, previous research shows that the higher incidence of cancer among males is only partially explained by non-biological risk factors by statistically adjusting for behaviors (e.g., smoking, alcohol use), lifestyle (e.g., physical activity, diet, medications), and comorbidities that may differ between men and women [4, 5, 7]. A global ecological analysis concluded that the sex difference for most cancer sites examined was partially or entirely unexplained by known risk factors [4]. Another analysis that quantified the sex difference explained by non-biological factors in a US cohort study found only a modest proportion of the male excess was explained by risk factors and differed by cancer site [5].

The extent to which external exposures explain the male predominance of cancer could be differential by race and ethnicity [8–11]. Prior studies were unable to account for risk factors with varying prevalence between the sexes by race and ethnicity [5, 12]. For instance, the prevalence of smoking among Asian Americans is low overall (~ 9%), but Asian American men are 2–3 times more likely to smoke compared to Asian American women (e.g. men 15%, women 5%) [13, 14]. Conversely, there is little difference in the prevalence of smoking among non-Hispanic White men and women (men 21%, women 18%) [13]. Similarly, American women have healthier diets than men [15, 16] though this also differs by race and ethnicity with Hispanic individuals shown to have better quality diets than Black and White individuals [16]. However, previous studies on sex differences in cancer incidence either did not look at racial and ethnic differences or were conducted in majority White populations [3, 5, 7, 17, 18]. One study that did examine sex differences in cancer incidence by race and ethnicity found little variation in the male-to-female incidence rate ratio across racial and ethnic groups, however the authors were not able to control for cancer risk factors [12]. Using a racially and ethnically diverse cohort, we aim to examine whether cancer incidence in males compared to females differs by race and ethnicity, and to quantify how much of sex difference is statistically explained by cancer risk factors. Our hypothesis is that sex differences will remain across race/ethnicity, suggesting that differences in the sex ratio may be driven by intrinsic sex differences and not external factors.

## Materials and methods

The Multiethnic Cohort (MEC) Study is a prospective cohort study that enrolled men and women ages 45–75 years between 1993 and 1996 from Hawaii and California [19, 20]. MEC participants provided consent to participate, and the study was approved by the institutional review boards of the University of Hawaii and the University of Southern California.

A questionnaire was mailed to MEC participants to collect demographics, medical history, medication use, family history of cancer, smoking history, physical activity, and a food frequency questionnaire. Sex was self-reported as male or female. Race/ethnicity, a social construct (not genetic or biological) [21], was self-reported as Japanese American, Native Hawaiian, other Asian American/Pacific Islander (Chinese, Filipino, or Korean), Black/African American, Latino (Mexican or other Hispanic), and non-Hispanic White.

Cancer incidence was ascertained from linkages to state-wide cancer registries and deaths were identified from linkages to state death files and the National Death Index. Cancer incidence was defined as the first, primary cancer diagnosis after cohort entry, using the International Classification of Disease for Oncology (3rd edition) coding. We examined 13 solid tumor types with at least 50 incident cancers in each sex to ensure sufficient sample size [22]. These included cancers of the oral cavity, esophagus, stomach, colorectum, liver, gallbladder, pancreas, lung, skin (melanoma), kidney, bladder, brain, and thyroid (Supplemental Table 1) [23].

All covariates were collected via self-report on the baseline questionnaire. These included age (coded as continuous), place of birth (USA born or foreign born), maximum education obtained (≤ 8th grade, 9th–12th grade; some college or vocational school; and graduated college or attended graduate or professional school), body mass index (kg/m^2^), cigarettes (packs per year), smoking status (current, past, never), smoking pack-years (heavy smoker, ≥ 40 packyears; moderate smoker,≥20–<40 packyears; light smoker, < 20 packyears, or never smoker), occupation (laborer/farm worker; factory worker/machine operator; clerical/office worker; sales; manager/administrator; craftsperson; small business owner; professional/technical; unemployed; and other occupation), physical activity (quartiles of metabolic equivalent of task per day), medical history (diagnosis of diabetes, gallstones, high blood pressure, other skin cancer, polyps of intestines, partial removal of the stomach, ulcer – coded as yes or no), medication use (acetaminophen, antacid, aspirin use, pain relief medications, Tagamet, Zantac, or Pepcid – coded as yes, currently; yes, but not currently; or no), family history of cancer (including kidney, lung, skin, or stomach – coded as yes or no), dietary components (calories [Kcal/day]; added sugars [tsp/day]; alcoholic beverages (drinks/day) dairy, cheese [cups/day]; dairy, milk, including soy [cups/day]; dairy, total [cups/day]; dairy, yogurt [cups/day]; fruits, total, whole and juice [cups/day]; fruits, total, juice only [cups/day]; grains, total [oz/day]; meats, egg [ounces/day]; meats, fish, high-omega-3 [ounces/day]; meats, fish, low-omega-3 [ounces/day]; meats, frankfurters/sausage/luncheon meat [ounces/day]; meats, organ meats [ounces/day]; meats, poultry [ounces/day]; and vegetables, total [cups/day] – coded into quartiles), and consumption of multivitamins, with or without minerals (coded as yes or no).

There were over 215,000 individuals who completed the baseline questionnaire. We excluded 13 individuals with tumor diagnosis discrepancies. Individuals who did not identify as African American, Japanese American, Native Hawaiian, Latino, or White (*n* = 12,206) were also excluded due to small sample sizes of individual categories of self-identification. We further excluded individuals with a history of cancer, not including non-melanoma skin cancer (*n* = 17,794) and individuals who had implausible dietary values (*n* = 7,585) [24]. Our analysis included 178,036 individuals (81,664 males and 96,372 females).

### Statistical analyses

We conducted a descriptive analysis of the cohort using medians (interquartile ranges [IQR]) and counts (proportions) to summarize the demographic, health, and nutritional characteristics of the sample. We calculated the cancer incidence rates (IRs) per 100,000 person years by sex for each cancer site using Poisson regression. We used Cox proportional hazard regression to estimate the age-adjusted and fully adjusted male-to-female hazard ratio (MF HR) for each cancer site. Age was used as the time scale with follow-up time beginning at completion of the baseline questionnaire until cancer diagnosis, death or end of follow-up (December 31, 2017) [25]. We separately estimated MF HRs for each cancer site for each of the five major racial and ethnic groups. We then re-ran the Cox models separating the AAPI group into Japanese American and Native Hawaiian. Fully adjusted models controlled for external factors known to alter cancer risk (based on existing literature) for each cancer site (Supplemental Table 2). We included an interaction term for race and ethnicity by sex in the models to conduct a Wald test for the statistical significance of differing MF HRs across race and ethnicity. All fully adjusted models for each cancer site, except for brain cancer (likelihood ratio test *P* = 0.53) differed from the corresponding age-adjusted model (data not shown). The inclusion of education and marital status did not improve model fit, and these variables were not statistically significant, so they were removed from the final models. We tested the proportional hazards assumption for each model by adding a time-dependent covariate (sex by time-to-event) to each model and examining the Wald statistic [26]. There was no qualitative violation of the proportional hazards assumption.

To quantify the proportion of the sex differences statistically explained by external factors we used the Peters-Belson method for time-to-event survival outcomes [27]. We estimated the observed survival rate (1-survival= cancer incidence, per 100,000) for males (M_inc_) and for females (F_inc_) through 10 years of follow-up. Next, we developed a cancer site-specific Cox model in males and applied this model to females to predict the male counterfactual incidence rate if they had been females (C_inc)_). The Peters-Belson estimate for the male-to-female difference in cancer rate that is explained by differences in the covariate distributions between males and females was calculated as (M_inc_ – C_inc_)/(M_inc_ – F_inc_)*100. The resultant proportion is the percentage of male-to-female differences in cancer risk that is explained by differences in risk factor distributions between men and women. We calculated the overall and the race- or ethnic-specific proportions for each cancer site. Japanese American and Native Hawaiian were combined due to small number of cancer events. A negative Peters-Belson estimate indicates that the males would have greater than observed incidence if they had the covariate distribution of women [28]. In the analyses by race or ethnicity, we removed the presentation of subgroup analyses where the case count was insufficient to produce stable estimates (e.g., < 50 cases).

We also conducted several sensitivity analyses. First, to inspect residual confounding, we estimated the MF HR in never and ever smokers, separately, for cancer sites where smoking is a risk factor which included cancers of the bladder, colorectum, esophagus, gallbladder, kidney, liver, oral cavity, pancreas, stomach and lung. Finally, to account for unmeasured confounding, we adjusted for hepatitis B (HBV) and hepatitis C virus (HCV) infections for liver cancer [29], using method described by Steenland and Greenland [30]. Briefly, we used the sex and race/ethnicity specific prevalence of HBV and HCV among US adults aged 50–70 years from the National Health and Nutrition Examination Survey III [31] to externally adjust our risk ratios.

All analyses were conducted using SAS version 9.4 (The SAS Institute, Carey, NC). We used complete case analysis. All *P*-values reported as two-sided.

## Results

The analysis included 178,036 individuals (81,664 males and 96,372 females), contributing to a total of 3,247,747.73 person-years with a mean of 17.4 person-years for males and a mean of 19.3 person-years for females. Table 1 displays the frequency and distribution of select demographic, health, and lifestyle factors by sex (additional factors listed in Supplemental Table 3). The median age for both males and females was 60 years (IQR, 52–67 years). The racial and ethnic breakdown for males was 13% African American, 30% Japanese American, 26% Latino, 24% White, and 7% Native Hawaiian, while the breakdown for females was 20% African American, 27% Japanese American, 23% Latino, 23% White, and 7% Native Hawaiian. Of the 32,264 (18%) participants that were born outside of the U.S., 45% were males while 55% were females.

**Table 1:** Distribution of demographic, medical history, and lifestyle information among participants in the Multiethnic Cohort Study by sex (1993–1996)

	Total *N* = 178,036 *N* (%)	Male *N* = 81,664 *N* (%)	Female *N* = 96,372 *N* (%)
**Demographics**			
Age: Median [Min, Max]	60.0 [45.0,78.0]	60.0 [45.0,78.0]	60.0 [45.0,78.0]
**Ethnicity**			
African American	30,029 (16.87)	10,944 (13.40)	19,085 (19.80)
Japanese American	50,596 (28.42)	24,340 (29.81)	26,256 (27.24)
Native Hawaiian and Other Asian American Pacific Islander^a^	12,804 (7.19)	5733 (7.02)	7071 (7.34)
Latino	42,608 (23.93)	20,891 (25.58)	21,717 (22.53)
White	41,999 (23.59)	19,756 (24.19)	22,243 (23.08)
**Marital status**			
Married	118,311 (66.97)	62,188 (76.65)	56,123 (58.74)
Separated	5270 (2.98)	1922 (2.37)	3348 (3.50)
Divorced	23,398 (13.24)	8235 (10.15)	15,163 (15.87)
Widowed	17,765 (10.06)	3027 (3.73)	14,738 (15.43)
Never married	11,787 (6.67)	5706 (7.03)	6081 (6.37)
Unknown	136 (0.08)	52 (0.06)	84 (0.09)
Missing	1369 (0.77)	534 (0.65)	835 (0.87)
**Place of birth**			
USA born	145,197 (81.82)	66,838 (82.05)	78,359 (81.62)
Foreign born	32,264 (18.18)	14,619 (17.95)	17,645 (18.38)
Missing	575 (0.32)	207 (0.25)	368 (0.38)
**Maximum education obtained**			
8th grade or less	20,299 (11.54)	9398 (11.64)	10,901 (11.46)
9th – 12th grade	58,260 (33.12)	24,453 (30.28)	33,807 (35.53)
Some college or vocational school	51,285 (29.15)	23,473 (29.06)	27,812 (29.23)
Graduated college or attended graduate or professional school	46,069 (26.19)	23,442 (29.02)	22,627 (23.78)
Missing	2123 (1.19)	898 (1.10)	1225 (1.27)
***Anthropometrics***			
Body mass index (kg/m^2^): Median [Min, Max]	25.88 [10.19, 81.81]	26.04 [10.87,71.56]	25.56 [10.19, 81.81]
***Lifestyle factors***			
**Cigarette packs per year: Median [Min**,** Max]**	1.25 [0, 63.55]	6.38 [0, 63.55]	0 [0, 63.55]
**Smoking status**			
Never smoker	77,439 (44.20)	24,283 (30.08)	53,156 (56.27)
Past	69,289 (39.55)	41,556 (51.48)	27,733 (29.36)
Current	28,458 (16.24)	14,879 (18.43)	13,579 (14.37)
Missing	2850 (1.60)	946 (1.16)	1904 (1.98)
**Pack-years**			
Never smoker	77,439 (45.21)	24,283 (30.92)	53,156 (57.31)
Light smoker (< 20 packyears)	64,560 (37.69)	34,423 (43.83)	30,137 (32.49)
Moderate smoker (≥20–<40 packyears)	19,274 (11.25)	12,675 (16.14)	6599 (7.11)
Heavy smoker (≥40 packyears)	10,027 (5.85)	7163 (9.12)	2864 (3.09)
Missing	6736 (3.78)	3120 (3.82)	3616 (3.75)
***Medical history***			
**Diabetes**			
No	157,339 (88.37)	71,524 (87.58)	85,815 (89.05)
Yes	20,697 (11.63)	10,140 (12.42)	10,557 (10.95)
**High blood pressure**			
No	109,102 (61.28)	49,373 (60.46)	59,729 (61.98)
Yes	68,934 (38.72)	291 (39.54)	36,643 (38.02)
***Occupation***			
Laborer/farm worker	6884 (3.87)	4995 (6.11)	1889 (1.96)
Factory worker/machine operator	19,860 (11.16)	10,915 (13.37)	8945 (9.28)
Clerical/office worker	29,216 (16.41)	4271 (5.23)	24,945 (25.88)
Sales	9332 (5.24)	3817 (4.67)	5515 (5.72)
Manager/Administrator	15,901 (8.93)	10,324 (12.64)	5577 (5.79)
Craftsperson	6474 (3.64)	5734 (7.02)	740 (0.77)
Small business owner	9871 (5.55)	5864 (7.18)	4007 (4.16)
Professional/technical	39,325 (22.10)	20,901 (25.59)	18,424 (19.11)
Unemployed	6324 (3.55)	913 (1.12)	5411 (5.61)
Other occupation	20,720 (11.64)	9086 (11.13)	11,634 (12.07)
Missing	30,030 (16.87)	15,168 (18.57)	14,862 (15.42)
***Physical activity***			
METS of activity for a typical 24-hour day relative to 1 for sitting: Median [Min, Max]	1.62 [0.96, 4.04]	1.64 [0.96, 4.04]	1.60 [0.96, 4.04]
***Cancer Incidence Site***			
No cancer	133,439 (75.81)	57,811 (71.75)	75,628 (79.23)
Oral cavity	479 (0.27)	292 (0.36)	187 (0.20)
Esophagus	343 (0.19)	268 (0.33)	75 (0.08)
Stomach	1,341 (0.76)	777 (0.96)	564 (0.59)
Colon	3,674 (2.09)	1,766 (2.19)	1,908 (2.00)
Rectum	1,221 (0.69)	752 (0.93)	469 (0.49)
Liver	902 (0.51)	562 (0.70)	340 (0.36)
Gallbladder	153 (0.09)	51 (0.06)	102 (0.11)
Pancreas	1,531 (0.87)	691 (0.86)	840 (0.88)
Lung	4,965 (2.82)	2,695 (3.34)	2,270 (2.38)
Melanoma	962 (0.55)	581 (0.72)	381 (0.40)
Kidney	1,081 (0.61)	633 (0.79)	448 (0.47)
Bladder	898 (0.51)	656 (0.81)	242 (0.25)
Brain	304 (0.17)	154 (0.19)	150 (0.16)
Thyroid	396 (0.22)	102 (0.13)	294 (0.31)
Missing	2,008 (1.13)	1,091 (1.34)	917 (0.95)

kg/m2, kilograms per meters squared; max, maximum; METS, metabolic equivalent; min, minimum,N, number

a Includes individuals who identified with Asian American/Pacific Islander, Chinese, Filipino, or Korean ethnicities

Table 2 displays the unadjusted IRs for males and females along with the age-adjusted MF HRs and fully adjusted MF HRs. The cancers with the highest incidence were lung (male-IR 191.7, 95% CI 179.4-205.0, female-IR 123.2, 95% CI 114.6-132.5) and colorectal (Male IR 179.2, 95% CI 166.1-193.3, Female IR 129.0, 95% CI 119.4-139.5). In comparison to the age-adjusted MF HR, the fully adjusted MF HRs were slightly attenuated when controlling for known risk cancer factors. However, there remained higher male incidence for cancers of the esophagus, bladder, liver, oral cavity, skin, kidney, stomach, lung, colorectal, brain, and pancreas, with MF HRs ranging from 1.09 (95% CI 1.03, 1.16) for lung to 3.60 (95% CI 2.68–4.84) for esophageal, while the smaller MF HRs remained for cancers of the gallbladder (MF HR 0.61, 95% CI 0.42–0.90) and thyroid (MF HR 0.45, 95% CI 0.35–0.57).

**Table 2:** Unadjusted cancer incidence rates by sex, age-adjusted male-to-female hazard ratios, fully adjusted male-to-female hazard ratios, and proportion explained by cancer risk factors

Cancer Site	Unadjusted Male IR, per 100,000 py (95% CI)	Unadjusted Female IR, per 100,000 py (95% CI)	Age-Adjusted MF HR (95% CI)	Fully adjusted MF HR (95% CI)	Proportion Explained^*n*^
Oral cavity	20.8 (16.3, 26.5)	10.2 (7.5, 13.7)	2.09 (1.74, 2.51)	1.52 (1.22, 1.90) ^*a*^	49.0%
Esophageal	19.1 (15.5, 23.4)	4.1 (2.8, 6.0)	4.87 (3.77, 6.30)	3.60 (2.68, 4.84) ^*b*^	30.7%
Stomach	55.3 (48.6, 62.9)	30.6 (26.3, 35.6)	1.90 (1.70, 2.12)	1.57 (1.37, 1.79) ^*c*^	26.7%
Colorectal	179.2 (166.1, 193.3)	129.0 (119.4, 139.5)	1.43 (1.35, 1.51)	1.26 (1.18, 1.34) ^*d*^	31.7%
Liver	40.0 (35.2, 45.5)	18.5 (15.6, 21.8)	2.28 (2.00, 2.61)	1.79 (1.54, 2.09) ^*e*^	23.8%
Gallbladder	3.6 (2.2, 5.9)	5.5 (3.9, 7.8)	0.69 (0.49, 0.97)	0.61 (0.42, 0.90) ^*f*^	0.0%
Pancreas	49.2 (43.5, 55.5)	45.6 (40.8, 50.9)	1.15 (1.04, 1.27)	1.14 (1.01, 1.29) ^*g*^	11.6%
Lung	191.7 (179.4, 205.0)	123.2 (114.6, 132.5)	1.63 (1.54, 1.72)	1.09 (1.03, 1.16) ^*h*^	81.9%
Melanoma	41.3 (36.1, 47.4)	20.7 (17.5, 24.5)	2.05 (1.80, 2.34)	1.93 (1.67, 2.20) ^*i*^	-10.1%
Kidney	45.0 (39.4, 51.5)	24.3 (20.8, 28.5)	1.90 (1.69, 2.15)	1.82 (1.57, 2.10) ^*j*^	8.2%
Bladder	46.7 (41.1, 53.1)	13.1 (10.6, 16.2)	3.80 (3.28, 4.41)	3.00 (2.55, 3.52) ^*k*^	30.0%
Brain	11.0 (8.3, 14.5)	8.1 (6.1, 10.9)	1.39 (1.11, 1.74)	1.36 (1.08, 1.72) ^*l*^	2.7%
Thyroid	7.3 (5.0, 10.5)	16.0 (12.8, 19.9)	0.46 (0.36, 0.57)	0.45 (0.35, 0.57) ^*m*^	6.9%

CI, confidence interval; IR, incidence ratio; MF HR, male-to-female hazard ratio

^*a*^Adjusted for age (continuous), occupation (laborer/farm worker/factory worker/machine operator, clerical/office worker/sales/manager/administrator, craftsperson/small business owner/professional/technical, unemployed/other occupation or missing), BMI (normal, underweight, overweight, obese), smoking status (never smoker, past, current), smoking packyears (never smoker, light smoker, moderate smoker, heavy smoker), alcohol use (no drinking, low to moderate drinking, heavy drinking), physical activity (sedentary, low to moderate activity, vigorous activity), calorie intake (25^th^,50^th^, 75^th^and 100^th^ quartile, kcal/day), sugar intake (25th,50th, 75th and 100th quartile, tsp/day), dairy intake (25th,50th, 75th and 100th quartile, cups/day), total fruit intake (25th,50th, 75th and 100th quartile, cups/day), total fruit juice intake (25th,50th, 75th and 100th quartile, cups/day), total grains intake (25th,50th, 75th and 100th quartile, cups/day), all meat intake-including poultry and fish (25th,50th, 75th and 100th quartile), ounces/day), total vegetable intake (25th,50th, 75th and 100th quartile, cups/day), fish with low omega-3 (25th,50th, 75th and 100th quartile, ounces/day), fish with high omega-3 (25th,50th, 75th and 100th quartile, ounces/day), processed meat intake (25^th^,50^th^, 75^th^and 100^th^ quartile, ounces/day) and meat intake (25^th^,50^th^, 75^th^and 100^th^ quartile, ounces/day)

^*b*^Adjusted for age (continuous), BMI (normal, underweight, overweight, obese), smoking status (never smoker, past, current), smoking packyears(never smoker, light smoker, moderate smoker, heavy smoker), physical activity (sedentary, low to moderate activity, vigorous activity), alcohol use (no drinking, low to moderate drinking, heavy drinking), antacid use (no, yes-but not currently, yes-currently), and Tagamet, Zantac, or Pepcid use (no, yes-but not currently, yes-currently)

^*c*^Adjusted for age (continuous), race/ethnicity (African American, Japanese American, Native Hawaiian, Latino, White), place of birth (US born or foreign born), occupation (laborer/farm worker/factory worker/machine operator, clerical/office worker/sales/manager/administrator, craftsperson/small business owner/professional/technical, unemployed/other occupation or missing), BMI (normal, underweight, overweight, obese), history of polyps of intestines (yes or no), history of partial removal of the stomach (yes or no), family history of stomach cancer (yes or no), history of high blood pressure (yes or no), total fruit intake (25th,50th, 75th and 100th quartile, cups/day), total fruit juice intake (25th,50th, 75th and 100th quartile, cups/day), total grain intake (25th,50th, 75th and 100th quartile, cups/day), all meat intake-including poultry and fish (25th,50th, 75th and 100th quartile), total vegetable intake (25th,50th, 75th and 100th quartile, cups/day), fish with low omega-3 (25th,50th, 75th and 100th quartile, ounces/day), fish with high omega-3 (25th,50th, 75th and 100th quartile, ounces/day), processed meat intake (25^th^,50^th^, 75^th^and 100^th^ quartile, ounces/day) and meat intake (25^th^,50^th^, 75^th^and 100^th^ quartile, ounces/day), alcohol use (no drinking, low to moderate drinking, heavy drinking), smoking status (never smoker, past, current), smoking packyears(never smoker, light smoker, moderate smoker, heavy smoker), and history of ulcers (yes or no)

^*d*^Adjusted for age (continuous), race/ethnicity (African American, Japanese American, Native Hawaiian, Latino, White), history of polyps of intestines (yes or no), BMI (normal, underweight, overweight, obese), physical activity (sedentary, low to moderate activity, vigorous activity),  calorie intake (25^th^,50^th^, 75^th^and 100^th^ quartile, kcal/day), alcohol use (no drinking, low to moderate drinking, heavy drinking), sugar intake (25th,50th, 75th and 100th quartile, tsp/day), smoking status (never smoker, past, current), smoking packyears(never smoker, light smoker, moderate smoker, heavy smoker), processed meat intake (25^th^,50^th^, 75^th^and 100^th^ quartile, ounces/day), meat intake (25^th^,50^th^, 75^th^and 100^th^ quartile, ounces/day), organ meat intake (less than 1 ounce/day or 1+ ounces/day) and consumption of multivitamins (yes or no)

^*e*^Adjusted for age (continuous), history of diabetes (yes or no), BMI (normal, underweight, overweight, obese), alcohol use (no drinking, low to moderate drinking, heavy drinking), race/ethnicity (African American, Japanese American, Native Hawaiian, Latino, White),sugar intake (25th,50th, 75th and 100th quartile, tsp/day), and smoking status (never smoker, past, current), smoking packyears(never smoker, light smoker, moderate smoker, heavy smoker)

^*f*^Adjusted for age (continuous), race/ethnicity (African American, Japanese American, Native Hawaiian, Latino, White), history of gallstones (yes or no), occupation (laborer/farm worker/factory worker/machine operator, clerical/office worker/sales/manager/administrator, craftsperson/small business owner/professional/technical, unemployed/other occupation or missing), BMI (normal, underweight, overweight, obese), and smoking status (never smoker, past, current), smoking packyears(never smoker, light smoker, moderate smoker, heavy smoker)

^*g*^Adjusted for age (continuous), race/ethnicity (African American, Japanese American, Native Hawaiian, Latino, White), occupation (laborer/farm worker/factory worker/machine operator, clerical/office worker/sales/manager/administrator, craftsperson/small business owner/professional/technical, unemployed/other occupation or missing), smoking status (never smoker, past, current), smoking packyears (never smoker, light smoker, moderate smoker, heavy smoker), alcohol use (no drinking, low to moderate drinking, heavy drinking), physical activity (sedentary, low to moderate activity, vigorous activity), calorie intake (25^th^,50^th^, 75^th^and 100^th^ quartile, kcal/day), sugar intake (25th,50th, 75th and 100th quartile, tsp/day), history of diabetes (yes or no), processed meat intake (25^th^,50^th^, 75^th^and 100^th^ quartile, ounces/day), meat intake (25^th^,50^th^, 75^th^and 100^th^ quartile, ounces/day), and family history of pancreatic cancer (yes or no)

^*h*^Adjusted for age (continuous), race/ethnicity (African American, Japanese American, Native Hawaiian, Latino, White),family history of lung cancer (yes or no), place of birth (US born or foreign born), smoking status (never smoker, past, current), smoking packyears (never smoker, light smoker, moderate smoker, heavy smoker) and occupation (laborer/farm worker/factory worker/machine operator, clerical/office worker/sales/manager/administrator, craftsperson/small business owner/professional/technical, unemployed/other occupation or missing)

^*i*^Adjusted for age (continuous), race/ethnicity (African American, Japanese American, Native Hawaiian, Latino, White), occupation (laborer/farm worker/factory worker/machine operator, clerical/office worker/sales/manager/administrator, craftsperson/small business owner/professional/technical, unemployed/other occupation or missing), and family history of melanoma (yes or no)

^*j*^Adjusted for age (continuous), race/ethnicity (African American, Japanese American, Native Hawaiian, Latino, White), family history of kidney cancer (yes or no), BMI (normal, underweight, overweight, obese), smoking status (never smoker, past, current), smoking packyears (never smoker, light smoker, moderate smoker, heavy smoker), occupation (laborer/farm worker/factory worker/machine operator, clerical/office worker/sales/manager/administrator, craftsperson/small business owner/professional/technical, unemployed/other occupation or missing), history of high blood pressure (yes or no),  acetaminophen use (no, yes-but not currently, yes-currently), and aspirin use (no, yes-but not currently, yes-currently)

^*k*^Adjusted for age (continuous), race/ethnicity (African American, Japanese American, Native Hawaiian, Latino, White), place of birth (US born or foreign born), smoking status (never smoker, past, current), smoking packyears (never smoker, light smoker, moderate smoker, heavy smoker), history of diabetes (yes or no) and occupation (laborer/farm worker/factory worker/machine operator, clerical/office worker/sales/manager/administrator, craftsperson/small business owner/professional/technical, unemployed/other occupation or missing)

^*l*^Adjusted for Adjusted for age (continuous) and occupation (laborer/farm worker/factory worker/machine operator, clerical/office worker/sales/manager/administrator, craftsperson/small business owner/professional/technical, unemployed/other occupation or missing)

^*m*^Adjusted for age (continuous), occupation (laborer/farm worker/factory worker/machine operator, clerical/office worker/sales/manager/administrator, craftsperson/small business owner/professional/technical, unemployed/other occupation or missing), BMI (normal, underweight, overweight, obese), place of birth (US born or foreign born), fish with low omega-3 (25th,50th, 75th and 100th quartile, ounces/day), fish with high omega-3 (25th,50th, 75th and 100th quartile, ounces/day), cheese intake (25^th^,50^th^, 75^th^and 100^th^ quartile, cups/day), milk intake (25^th^,50^th^, 75^th^and 100^th^ quartile, cups/day), dairy intake (25^th^,50^th^, 75^th^and 100^th^ quartile, cups/day), yogurt intake (less than 1 ounce/day or 1+ cups/day) and egg intake (25^th^,50^th^, 75^th^and 100^th^ quartile, ounces/day)

^*n*^Estimated using the Peters-Belson method

The Peters-Belson estimates quantifying the proportion of the external factors that explain the observed sex difference in incidence are presented in Table 2. The proportions differed widely, e.g., 0% explained by the external factors (age, history of gallstones, race and ethnicity, occupation, and smoking packyears) for gallbladder cancer, 82% explained by the external factors for lung cancer incidence (age, family history of lung cancer, place of birth, smoking packyears, race and ethnicity, and occupation), and − 10% was explained by the adjusted factors (age, occupation, and race and ethnicity) for melanoma, which meant if men had the covariate distribution of women, the observed male incidence would have been higher.

Table 3 shows the fully adjusted MF HRs by race and ethnicity. For most cancers, the MF HR did not differ significantly across the five racial and ethnic categories. However, the MF HR differed across race and ethnicity for cancers of the esophagus (*P* = 0.018), liver (*P* = 0.005) and thyroid (*P* = 0.005). Among those cancers with significant interaction, we found the strongest MF HRs for: (1) esophageal cancer for Native Hawaiian (MF HR 17.67, 95% CI: 2.21, 141.20) and Latino individuals (MF HR 6.33, 95% CI 3.00-13.33) (2) liver cancer for African American (MF HR 2.52, 95% CI 1.67–3.79) and White individuals (MF HR 2.79, 95% CI 1.81–4.30). Thyroid cancer had a female predominance among all race and ethnic groups with the strongest sex difference seen amongst Latino individuals (MF HR 0.21, 95% CI 0.13–0.35). The Peters-Belson estimates quantifying the proportion of the external factors that explain the observed sex difference in incidence by race and ethnicity are provided in Supplemental Table 4.

**Table 3:** Fully adjusted male-to-female hazard ratios for each cancer site by race and ethnicity

Cancer Site	African American	Japanese American	Native Hawaiian	Latino	White	Interaction *P*-values ^a^
	MF HR (95% CI)	MF HR (95% CI)	MF HR (95% CI)	MF HR (95% CI)	MF HR (95% CI)	
Oral Cavity ^*b*^	0.89 (0.47, 1.67)	1.17 (0.76, 1.79)	2.52 (1.04, 6.13)	1.81 (1.10, 2.99)	1.92 (1.27, 2.91)	0.250
Esophageal ^*c*^	1.28 (0.69, 2.36)	5.18 (2.43, 11.06)	17.67 (2.21, 141.20)	6.33 (3.00, 13.33)	3.43 (2.03, 5.81)	0.018
Stomach ^*d*^	1.23 (0.86, 1.74)	1.70 (1.37, 2.11)	2.04 (1.25, 3.32)	1.47 (1.13, 1.93)	1.63 (1.08, 2.45)	0.424
Colorectal ^*e*^	1.24 (1.06, 1.45)	1.29 (1.14, 1.45)	1.21 (0.94, 1.57)	1.26 (1.08, 1.46)	1.19 (1.02, 1.37)	0.463
Liver ^*f*^	2.52 (1.67, 3.79)	1.28 (0.96, 1.70)	2.43 (1.32, 4.47)	1.55 (1.19, 2.01)	2.79 (1.81, 4.30)	0.005
Gallbladder ^*g*^	0.69 (0.24, 1.98)	0.97 (0.41, 2.32)	1.24 (0.33, 4.71)	0.35 (0.19, 0.65)	0.73 (0.29, 1.88)	0.099
Pancreas ^*h*^	0.98 (0.74, 1.29)	1.08 (0.87, 1.35)	1.23 (0.83, 1.84)	1.19 (0.90, 1.56)	1.29 (0.98, 1.68)	0.595
Lung ^*i*^	1.15 (1.02, 1.31)	1.19 (1.04, 1.36)	1.25 (1.04, 1.52)	1.02 (0.87, 1.21)	0.98 (0.87, 1.10)	0.372
Melanoma ^j^	3.53 (0.82, 15.18)	1.04 (0.56, 1.93)	3.94 (1.89, 8.18)	1.89 (1.13, 3.17)	1.93 (1.67, 2.23)	0.146
Kidney ^*k*^	1.63 (1.14, 2.34)	1.55 (1.15, 2.07)	2.29 (1.44, 3.66)	1.71 (1.31, 2.23)	2.17 (1.58, 2.98)	0.882
Bladder ^*l*^	2.47 (1.71, 3.56)	3.03 (2.17, 4.21)	2.43 (1.36, 4.34)	3.97 (2.59, 6.08)	3.35 (2.52, 4.46)	0.258
Brain ^*m*^	1.44 (0.72, 2.89)	1.19 (0.69, 2.06)	1.98 (0.86, 4.55)	1.20 (0.80, 1.82)	1.37 (0.91, 2.08)	0.980
Thyroid ^*n*^	0.55 (0.27, 1.11)	0.44 (0.27, 0.70)	0.66 (0.34, 1.28)	0.21 (0.13, 0.35)	0.83 (0.50, 1.37)	0.005

CI, confidence interval and MF HR, male-to-female hazard ratio

^*a*^P-value from Wald test assessing the statistical significance of the estimated interaction term for sex by race/ethnicity in the Cox proportional hazards model

^*b*^Adjusted for age (continuous), occupation (laborer/farm worker/factory worker/machine operator, clerical/office worker/sales/manager/administrator, craftsperson/small business owner/professional/technical, unemployed/other occupation or missing), BMI (normal, underweight, overweight, obese), smoking status (never smoker, past, current), smoking packyears (never smoker, light smoker, moderate smoker, heavy smoker), alcohol use (no drinking, low to moderate drinking, heavy drinking), physical activity (sedentary, low to moderate activity, vigorous activity), calorie intake (25^th^,50^th^, 75^th^and 100^th^ quartile, kcal/day), sugar intake (25th,50th, 75th and 100th quartile, tsp/day), dairy intake (25th,50th, 75th and 100th quartile, cups/day), total fruit intake (25th,50th, 75th and 100th quartile, cups/day), total fruit juice intake (25th,50th, 75th and 100th quartile, cups/day), total grains intake (25th,50th, 75th and 100th quartile, cups/day), all meat intake-including poultry and fish (25th,50th, 75th and 100th quartile), ounces/day), total vegetable intake (25th,50th, 75th and 100th quartile, cups/day), fish with low omega-3 (25th,50th, 75th and 100th quartile, ounces/day), fish with high omega-3 (25th,50th, 75th and 100th quartile, ounces/day), processed meat intake (25^th^,50^th^, 75^th^and 100^th^ quartile, ounces/day) and meat intake (25^th^,50^th^, 75^th^and 100^th^ quartile, ounces/day)

^*c*^Adjusted for age (continuous), BMI (normal, underweight, overweight, obese), smoking status (never smoker, past, current), smoking packyears (never smoker, light smoker, moderate smoker, heavy smoker), physical activity (sedentary, low to moderate activity, vigorous activity), alcohol use (no drinking, low to moderate drinking, heavy drinking), antacid use (no, yes-but not currently, yes-currently), and Tagamet, Zantac, or Pepcid use (no, yes-but not currently, yes-currently)

^*d*^Adjusted for age (continuous), place of birth (US born or foreign born), occupation (laborer/farm worker/factory worker/machine operator, clerical/office worker/sales/manager/administrator, craftsperson/small business owner/professional/technical, unemployed/other occupation or missing), BMI (normal, underweight, overweight, obese), history of polyps of intestines (yes or no), history of partial removal of the stomach (yes or no), family history of stomach cancer (yes or no), history of high blood pressure (yes or no), total fruit intake (25th,50th, 75th and 100th quartile, cups/day), total fruit juice intake (25th,50th, 75th and 100th quartile, cups/day), total grain intake (25th,50th, 75th and 100th quartile, cups/day), all meat intake-including poultry and fish (25th,50th, 75th and 100th quartile), total vegetable intake (25th,50th, 75th and 100th quartile, cups/day), fish with low omega-3 (25th,50th, 75th and 100th quartile, ounces/day), fish with high omega-3 (25th,50th, 75th and 100th quartile, ounces/day), processed meat intake (25^th^,50^th^, 75^th^and 100^th^ quartile, ounces/day) and meat intake (25^th^,50^th^, 75^th^and 100^th^ quartile, ounces/day), alcohol use (no drinking, low to moderate drinking, heavy drinking), smoking status (never smoker, past, current), smoking packyears (never smoker, light smoker, moderate smoker, heavy smoker) and history of ulcers (yes or no)

^*e*^Adjusted for age (continuous), history of polyps of intestines (yes or no), BMI (normal, underweight, overweight, obese), physical activity (sedentary, low to moderate activity, vigorous activity),  calorie intake (25^th^,50^th^, 75^th^and 100^th^ quartile, kcal/day), alcohol use (no drinking, low to moderate drinking, heavy drinking), sugar intake (25th,50th, 75th and 100th quartile, tsp/day), smoking status (never smoker, past, current), smoking packyears (never smoker, light smoker, moderate smoker, heavy smoker), processed meat intake (25^th^,50^th^, 75^th^and 100^th^ quartile, ounces/day), meat intake (25^th^,50^th^, 75^th^and 100^th^ quartile, ounces/day), organ meat intake (less than 1 ounce/day or 1+ ounces/day) and consumption of multivitamins (yes or no)

^*f*^Adjusted for age (continuous), history of diabetes (yes or no), BMI (normal, underweight, overweight, obese), alcohol use (no drinking, low to moderate drinking, heavy drinking), sugar intake (25th,50th, 75th and 100th quartile, tsp/day and smoking status (never smoker, past, current), and smoking packyears (never smoker, light smoker, moderate smoker, heavy smoker)

^*g*^Adjusted for age (continuous), history of gallstones (yes or no), occupation (laborer/farm worker/factory worker/machine operator, clerical/office worker/sales/manager/administrator, craftsperson/small business owner/professional/technical, unemployed/other occupation or missing), BMI (normal, underweight, overweight, obese), and smoking status (never smoker, past, current), and smoking packyears (never smoker, light smoker, moderate smoker, heavy smoker)

^*h*^Adjusted for age (continuous), occupation (laborer/farm worker/factory worker/machine operator, clerical/office worker/sales/manager/administrator, craftsperson/small business owner/professional/technical, unemployed/other occupation or missing), smoking status (never smoker, past, current), smoking packyears (never smoker, light smoker, moderate smoker, heavy smoker), alcohol use (no drinking, low to moderate drinking, heavy drinking), physical activity (sedentary, low to moderate activity, vigorous activity), calorie intake (25^th^,50^th^, 75^th^and 100^th^ quartile, kcal/day), sugar intake (25th,50th, 75th and 100th quartile, tsp/day), history of diabetes (yes or no), processed meat intake (25^th^,50^th^, 75^th^and 100^th^ quartile, ounces/day), meat intake (25^th^,50^th^, 75^th^and 100^th^ quartile, ounces/day), family history of pancreatic cancer (yes or no)

^*i*^Adjusted for age (continuous), family history of lung cancer (yes or no), place of birth (US born or foreign born), smoking status (never smoker, past, current), smoking packyears (never smoker, light smoker, moderate smoker, heavy smoker) and occupation (laborer/farm worker/factory worker/machine operator, clerical/office worker/sales/manager/administrator, craftsperson/small business owner/professional/technical, unemployed/other occupation or missing)

^*j*^Adjusted for age (continuous), occupation (laborer/farm worker/factory worker/machine operator, clerical/office worker/sales/manager/administrator, craftsperson/small business owner/professional/technical, unemployed/other occupation or missing), family history of melanoma (yes or no)

^*k*^Adjusted for age (continuous), family history of kidney cancer (yes or no), BMI (normal, underweight, overweight, obese), smoking status (never smoker, past, current), smoking packyears (never smoker, light smoker, moderate smoker, heavy smoker), occupation (laborer/farm worker/factory worker/machine operator, clerical/office worker/sales/manager/administrator, craftsperson/small business owner/professional/technical, unemployed/other occupation or missing), history of high blood pressure (yes or no),  acetaminophen use (no, yes-but not currently, yes-currently),  aspirin use (no, yes-but not currently, yes-currently)

^*l*^Adjusted for age (continuous), place of birth (US born or foreign born), smoking status (never smoker, past, current), smoking packyears (never smoker, light smoker, moderate smoker, heavy smoker), history of diabetes (yes or no) and occupation (laborer/farm worker/factory worker/machine operator, clerical/office worker/sales/manager/administrator, craftsperson/small business owner/professional/technical, unemployed/other occupation or missing)

^*m*^Adjusted for Adjusted for age (continuous) and occupation (laborer/farm worker/factory worker/machine operator, clerical/office worker/sales/manager/administrator, craftsperson/small business owner/professional/technical, unemployed/other occupation or missing)

^*n*^Adjusted for age (continuous), occupation (laborer/farm worker/factory worker/machine operator, clerical/office worker/sales/manager/administrator, craftsperson/small business owner/professional/technical, unemployed/other occupation or missing), BMI (normal, underweight, overweight, obese), place of birth (US born or foreign born), fish with low omega-3 (25th,50th, 75th and 100th quartile, ounces/day), fish with high omega-3 (25th,50th, 75th and 100th quartile, ounces/day), cheese intake (25^th^,50^th^, 75^th^and 100^th^ quartile, cups/day), milk intake (25^th^,50^th^, 75^th^and 100^th^ quartile, cups/day), dairy intake (25^th^,50^th^, 75^th^and 100^th^ quartile, cups/day), yogurt intake (less than 1 ounce/day or 1+ cups/day) and egg intake (25^th^,50^th^, 75^th^and 100^th^ quartile, ounces/day)

The results of the sensitivity analysis comparing the MF HRs comparing never smokers to ever smokers for cancers of the bladder, colorectal, re esophagus, gallbladder, kidney, liver, lung, oral cavity, pancreas, and stomach, are presented in Supplemental Table 5. The MF HR for liver cancer was larger among ever smokers compared to never smokers (ever smokers MF HR 2.33, 95% CI 1.94–2.79; never smokers MF HR 1.39, 95% CI 1.07–1.81, *P* = 0.027). When analyzed separately by race and ethnicity, the MF HR did not differ among the groups for any cancer site among never smokers only (*P* > 0.05) (Supplemental Table 6). Among ever smokers, the MF HRs differed significantly by race and ethnicity for esophageal cancer (*P* = 0.008) (Supplemental Table 7). For esophageal cancer, the male predominance disappeared among African American smokers (MF HR: 0.99, 95% CI: 0.51, 1.92), but remained elevated for Japanese American (MF HR 5.46, 95% CI: 1.93–15.41), Native Hawaiian (MF HR 14.44, 95% CI: 1.77-117.62), Latino (MF HR: 7.94, 95% CI: 2.82, 22.39), and White (MF HR 3.10, 95% CI 1.70, 5.63) smokers.

The results of the probabilistic sensitivity analysis accounting for unmeasured hepatitis B and C viruses are shown in Supplemental Table 8. Adjusting for the prevalence of hepatitis in the US population by race and ethnicity and sex attenuated the MF HR for liver cancer slightly, however, the elevated male incidence in liver cancer remained elevated across race and ethnicity.

## Discussion

Differential exposure to external factors namong males and females is often the presumed explanation for the higher male cancer incidence for most cancers at shared anatomic sites. Conversely, females had a higher risk of developing cancer of the gallbladder and thyroid. The cancer sites with the largest MF HRs after adjusting for multiple risk factors were esophagus and bladder cancers. These exposures can often differ by race and ethnicity due to geographical, cultural, and socioeconomic differences as well. However, we found after adjusting for many known cancer risk factors, the male predominance remained across race and ethnicity for cancers of the stomach, colorectum, liver, kidney, and bladder (MF HRs > 1.0 in all race/ethnic groups).

Across race and ethnicity, we saw differences in cancer risk between males and females for cancers of the esophagus, liver and thyroid (all *P*_*interaction*_<0.05). These findings suggest that there may be unaccounted for external exposure differences between men and women within these racial and ethnic groups. For instance, restricting these analyses to never smokers separately showed no sex differences by race and ethnicity for any cancer site, but we found a significant sex difference by race and ethnicity in esophageal cancer risk among ever smokers. These results indicate that there is likely residual confounding by smoking as it is unlikely that smoking affects the esophagus of one race or ethnicity differently from another. Smoking patterns and behaviors differ between men and women within these racial and ethnic groups [32], which highlights the need to stratify results by race and ethnicity as well as sex, and to examine external exposures among minority populations. Additionally, possible effect modifiers of the relationship between smoking and esophageal cancer, such as indoor and outdoor pollution, may differ in prevalence by race/ethnicity and between men and women [33, 34].

Our findings are similar to others who have examined sex difference in cancer incidence. Using SEER data, Cook et al. [3]. also found that esophageal and bladder cancer had the largest age-adjusted MF HRs. Edgren et al. [4]. found that accounting for cancer-specific risk factors neither completely nor partially explained the differences between males and females for cancers of the stomach, rectum, and brain, or for leukemia, lymphoma, and multiple myeloma, but partially explains the elevated male incidence in cancer incidence for esophagus, bladder, liver, kidney, skeleton and larynx. This study was an ecological study, and the authors were not able to quantify individual’s exposures. However, Jackson et al. [5]. showed that cancer-site-specific risk factors, including BMI and height, only partially explain sex differences in incidence. Similar to the present findings, that study found that only a modest proportion of the male excess cancer risk was explained by differential exposure to cancer risk factors (ranging from 80% for lung cancer to 11% for esophageal adenocarcinoma). Taken together, these studies indicate that the observed sex difference in cancer incidence is not entirely related to differential exposure distribution between men and women, but also likely related to intrinsic differences between the sexes or to risk factors that are sex specific.

Previous research posits that biological mechanisms such as sex steroid hormones and sex chromosome complement influence immune response resulting in the observed increased male cancer incidence [6, 35]. Sex hormones have been found to promote the growth of specific cancers and be a protective factor against other cancers [35]. For instance, estrogen growth-promoting characteristics have been linked to the development of thyroid cancer while higher levels of estrogen may reduce the risk of liver and colon cancer [36–40]. Furthermore, females tend to have a higher humoral response than males, including exhibiting higher levels of antibodies after infection [6, 40, 41]. Low humoral response among males may lead to chronic infection of oncogenic viruses which could promote tumorigenesis [42–46]. Sex chromosomes may also play a role in immune response and cancer progression. While one of the two X-chromosomes undergoes inactivation randomly, not all genes on the inactivated X are completely silenced. Tumor suppressor genes and other genes linked to immune response may escape X-chromosome inactivation, resulting in biallelic expression that may give females an immune advantage [47, 48].

There are many strengths to this study including the use of a large racially and ethnically diverse population. We were able to adjust for multiple external factors associated with cancer development such as age, lifestyle factors (smoking, alcohol use, diet, and physical activity level), occupation, and medical history. Some of our results should be interpreted with caution as we had small cell sizes in some subgroups. All exposures were measured by self-report, the accuracy of which could be differential by sex, and we may still have unmeasured risk factors or residual confounding. Though we externally adjusted for the population prevalence of viruses like hepatitis, our dataset lacked information on other oncogenic infections (e.g. human papillomavirus and Epstein-Barr virus) associated with some of the cancers we analyzed. We were also not able to adjust for environmental factors like ultraviolet radiation and chemical-specific occupational exposures that could have influenced the observed sex differences in cancer incidence. Finally, the sex differences in incidence rates may vary by histologic subtypes for some cancer sites, but we were limited by small sample sizes to examine this further.

In this comprehensive analysis, we observed that sex differences in cancer incidence persist after adjusting for multiple external factors. These sex differences persisted across race and ethnicity for most cancers. Only three cancer sites showed an interaction between race and ethnicity, indicating the influence of biologic explanations for the observed sex differences for most cancer sites. Future research should focus on intrinsic sex differences that may influence cancer incidence.

## Supplementary information

Supplementary material 1.

## Data Availability

The data that support the findings of this study are available from the Multiethnic Cohort Study (MEC) but restrictions apply to the availability of these data, which were used under license for the current study, and so are not publicly available. Data are, however, available from the authors upon reasonable request and with permission of MEC.
