# Supplementary material for: Sex differences in cancer incidence persist across race and ethnicity
Source: Biol Sex Differ. 2026 Apr 28;17:121. doi: 10.1186/s13293-026-00902-z (PMC13270759; doi:10.1186/s13293-026-00902-z)
Supplement: Supplementary file 1 — Supplementary material 1. [file 13293_2026_902_MOESM1_ESM.docx]

| **Supplemental Table 1.** International Classification of Disease for Oncology (ICD-O) coding for cancer incidence sites. | |
| --- | --- |
| **Cancer Incidence Site** | **ICD-O-3 Codes** |
| Oral Cavity | C00.0-C00.5, C01.9-C02.4, C02.8-C02.9, C03.0-C03.1, C03.9-C04.1, C04.9-C05.2, C05.8-C06.2, C06.8-C06.9, C07.9-C08.0, C08.9, C09.0-C09.1, C09.8-C10.3, C10.8-C10.9, C10.0, C11.0-C11.2, C11.8-C11.9, C12.9-C13.2, C13.8-C13.9, C14.0, C14.2, C14.8 |
| Esophagus | C15.0-C15.5, C15.8-C15.9 |
| Stomach | C16.0-C16.6, C16.8-C16.9 |
| Colorectal | C18.0-C18.9, C19.9, C20.9 |
| Liver | C22.0-C22.1 |
| Gallbladder | C23.9-C24.1, C24.8-C24.9 |
| Pancreas | C25.0-C25.4, C25.7-C25.9 |
| Lung | C34.0-C34.3, C34.8-C34.9 |
| Melanoma | C44.0-C44.9 |
| Kidney | C64.9 |
| Bladder | C67.0-C67.9 |
| Brain | C70.0, C70.9, C71.0-C71.9, C72.0-C72.1, C72.8-C72.9 |
| Thyroid | C73.9 |

| **Supplemental Table 2.** Cancer-specific adjustment factors for fully adjusted model. | | | |
| --- | --- | --- | --- |
| **Bladder**^1-6^   - - - - Age - Race/ethnicity - Place of Birth - Smoking Status - Pack-Years - History of Diabetes - Occupation | **Gallbladder**^7-10^   - Age - History of Gallstones - Race/ethnicity - Occupation - BMI - Smoking Status - Pack-Years | **Oral Cavity**^11^   - Age - Occupation - BMI - Smoking Status - Pack-Years - Alcohol Use - Physical Activity - Calorie Intake - Sugar Intake - Dairy Intake - Total Fruit Intake - Total Fruit Juice Intake - Total Grains Intake - All Meat Intake-Including Poultry and Fish - Total Vegetable Intake - Fish with Low Omega-3 - Fish with High Omega-3 - Processed Meat Intake - Meat Intake | **Lung**^12-14^   - Age - Family History of Lung Cancer - Place of Birth - Smoking Status - Pack-Years - Occupation |
| **Brain**^15,16^   - Age - Occupation | **Kidney**^17-20^   - Age - Family History of Kidney Cancer - BMI - Smoking Status - Pack-Years - Occupation - History of Hypertension - Acetaminophen Use - Aspirin Use - Race/ethnicity | **Pancreas**^21^   - Age - Occupation - Smoking Status - Pack-Years - Alcohol Use - Physical Activity - Calorie Intake - Sugar Intake - History of Diabetes - Processed Meat Intake - Meat Intake - Family History of Pancreatic Cancer - Race/ethnicity |  |
| **Colorectal**^22-25^   - Age - History of Polyps of Intestines - Race/ethnicity - BMI - Physical Activity - Calorie Intake - Alcohol Use - Sugar Intake - Smoking Status - Pack-Years - Processed Meat Intake - Meat Intake - Organ Meat Intake - Multivitamin Use | **Liver**^26-28^   - Age - History of Diabetes - BMI - Alcohol Use - Sugar Intake - Race/ethnicity - Smoking Status - Pack-Years | **Stomach**^29^   - Age - Race/ethnicity - Place Of Birth - Occupation - BMI - History Of Polyps of Intestines - History Of Partial Removal of The Stomach - Family History of Stomach Cancer - History Of Hypertension - Total Fruit Intake - Total Fruit Juice Intake - Total Grain Intake - All Meat Intake-Including Poultry and Fish - Total Vegetable Intake - Fish With Low Omega-3 - Fish With High Omega-3 - Processed Meat Intake - Meat Intake - Alcohol Use - Smoking Status - Pack-Years - History Of Ulcers |  |
| **Esophagus**^25,30,31^   - Age - BMI - Smoking - Pack-Years - Physical Activity - Alcohol Use - Antacid Use | **Melanoma**^32-34^   - Age - Occupation - Family History of Melanoma - Race/ethnicity | **Thyroid**^35^   - Age - Occupation - BMI - Place of Birth - Fish With Low Omega-3 - Fish With High Omega-3 - Cheese - Milk Intake - Dairy Intake - Yogurt Intake - Egg Intake |  |

| **Supplemental Table 3.** Distribution of medical history and dietary information among participants in the Multiethnic Cohort study by sex (1993-1996) | | | |
| --- | --- | --- | --- |
|  | **Total** N=178,036  N (%) | **Male** N=81,664  N (%) | **Female** N=96,372  N (%) |
| ***Medical history*** | | | |
| **Gallstones** | | | |
| No | 166,291 (93.40) | 78,679 (96.34) | 87,612 (90.91) |
| Yes | 11,745 (6.60) | 2,985(3.66) | 8,760 (9.09) |
| **Other skin cancer** | | | |
| No | 172,205 (96.72) | 78,755 (96.44) | 93,450 (96.97) |
| Yes | 5,831 (3.28) | 2,909 (3.56) | 2,922 (3.03) |
| **Polyps of intestines** | | | |
| No | 168,522 (94.66) | 76,228 (93.34) | 92,294 (95.77) |
| Yes | 9,514 (5.34) | 5,436 (6.66) | 4,078 (4.23) |
| **Partial removal of the stomach** | | | |
| No | 176,176 (98.96) | 80,590 (98.68) | 95,586 (99.18) |
| Yes | 1,860 (1.04) | 1,074 (1.32) | 786 (0.82) |
| **Ulcer** | | | |
| No | 157,270 (88.34) | 70,841 (86.75) | 86,429 (89.68) |
| Yes | 20,766 (11.66) | 10,823 (13.25) | 9,943 (10.32) |
| ***Medication History*** | | | |
| **Acetaminophen use** | | | |
| No | 115,080 (68.45) | 58,415 (75.06) | 56,665 (62.74) |
| Yes, but not currently | 29,442 (17.51) | 11,598 (14.90) | 17,844 (19.76) |
| Yes, currently | 23,609 (14.04) | 7,808 (10.03) | 15,801 (17.50) |
| Missing | 9,905 (5.56) | 3,843 (4.71) | 6,062 (6.29) |
| **Antacid use** | | | |
| No | 112,490 (66.34) | 54,167 (69.17) | 58,323 (63.91) |
| Yes, but not currently | 32,302 (19.05) | 14,245 (18.19) | 18,057 (19.79) |
| Yes, currently | 24,777 (14.61) | 9,897 (12.64) | 14,880 (16.31) |
| Missing | 8,467 (4.76) | 3,355 (4.11) | 5,112 (5.30) |
| **Aspirin use** | | | |
| No | 103,908 (60.82) | 46,671 (58.93) | 57,237 (62.46) |
| Yes, but not currently | 31,105 (18.21) | 13,669 (17.26) | 17,436 (19.03) |
| Yes, currently | 35,822 (20.97) | 18,856 (23.81) | 16,966 (18.51) |
| Missing | 7,201 (4.04) | 2,468 (3.02) | 4,733 (4.91) |
| **Pain relief medications use** | | | |
| No | 11,4642 (68.21) | 57,671 (74.24) | 56,971 (63.04) |
| Yes, but not currently | 31,707 (18.87) | 12,500 (16.09) | 19,207 (21.25) |
| Yes, currently | 21,711 (12.92) | 7,512 (9.67) | 14,199 (15.71) |
| Missing | 9,976 (5.60) | 3,981 (4.87) | 5,995 (6.22) |
| **Tagamet, Zantac, or Pepcid use** | | | |
| No | 147,335 (87.27) | 69,328 (88.78) | 78,007 (85.98) |
| Yes, but not currently | 13,770 (8.16) | 5,565 (7.13) | 82,05 (9.04) |
| Yes, currently | 7,714 (4.57) | 3,194 (4.09) | 4,520 (4.98) |
| Missing | 9,217 (5.18) | 3,577 (4.38) | 5,640 (5.85) |
| ***Family History of Cancer (in first degree relative)*** | | | |
| **Kidney cancer** | | | |
| No | 176,810 (99.31) | 81,160 (99.38) | 95,650 (99.25) |
| Yes | 1,226 (0.69) | 504 (0.62) | 722 (0.75) |
| **Lung cancer** | | | |
| No | 16,7163 (93.89) | 7,7162 (94.49) | 90,001 (93.39) |
| Yes | 10,873 (6.11) | 4,502 (5.51) | 6,371 (6.61) |
| **Melanoma** | | | |
| No | 177,279 (99.57) | 81,366 (99.64) | 95,913 (99.52) |
| Yes | 757 (0.43) | 298 (0.36) | 459 (0.48) |
| **Stomach cancer** | | | |
| No | 169,397 (95.15) | 77,766 (95.23) | 91,631 (95.08) |
| Yes | 8,639 (4.85) | 3,898 (4.77) | 4,741 (4.92) |
| ***Surveillance*** | | | |
| Age at death: Median [Min, Max] | 80.22 [73.12-86.42] | 79.44 [72.51-85.57] | 81.13 [73.86-87.29] |
| Age at cancer incidence diagnosis: Median [Min,Max] | 72.94 [66.62-78.88] | 73.09 [67.17-78.56] | 72.71 [65.89-79.25] |
| Age at exit: Median [Min,Max] | 77.88 [71.84-84.78] | 76.95 [71.28-83.50] | 78.72 [72.38-85.78] |
| ***Dietary Components*** | | | |
| Calories (Kcal/day) | 1956.88 [1445.45-2665.62] | 2198.72 [1641.48-2948.82] | 1771.16 [1323.47-2392.52] |
| Added sugars (tsp/day) | 8.79 [5.44-13.98] | 9.79 [6.05-15.58] | 8.03 [5.04-12.65] |
| Alcoholic beverages (drinks/day) | 0.01 [0.00-0.47] | 0.17 [0.00-1.21] | 0.00 [0.00-0.12] |
| Dairy, Cheese(cups/day) | 0.29 [0.15-0.54] | 0.31 [0.15-0.58] | 0.28 [0.14-0.51] |
| Dairy, Milk, include Soy (cups/day) | 0.51 [0.19-1.06] | 0.52 [0.20-1.07] | 0.50 [0.19-1.05] |
| Dairy, total (cups/day) | 0.98 [0.52-1.65] | 0.99 [0.52-1.67] | 0.97 [0.51-1.63] |
| Dairy, yogurt (cups/day) | 0.01 [0.00-0.07] | 0.00 [0.00-0.04] | 0.02 [0.00-0.08] |
| Fruits, Total, Whole+Juice (cups/day) | 1.70 [0.87-2.92] | 1.56 [0.79-2.73] | 1.82 [0.95-3.08] |
| Fruits, Total, Juice only (cups/day) | 0.14 [0.03-0.44] | 0.14 [0.03-0.44] | 0.14 [0.03-0.44] |
| Grains, Total (oz/day) | 7.00 [4.84-9.80] | 7.82 [5.50-10.89] | 6.35 [4.42-8.88] |
| Meats, Egg (oz/day) | 0.25 [0.14-0.45] | 0.31 [0.17-0.53] | 0.22 [0.12-0.39] |
| Meats, Fish, High-Omega-3 (oz/day) | 0.22 [0.01-0.43] | 0.27 [0.12-0.50] | 0.19 [0.08-0.38] |
| Meats, Fish, Low-Omega-3 (oz/day) | 0.30 [0.15-0.55] | 0.35 [0.17-0.63] | 0.27 [0.13-0.48] |
| Meats, Frankfurters/Sausage/Luncheon Meat (oz/day) | 0.30 [0.15-0.56] | 0.38 [0.20-0.68] | 0.25 [0.12-0.45] |
| Meats, Meat (oz/day) | 1.20 [0.65-2.02] | 1.49 [0.84-2.42] | 0.99 [0.54-1.67] |
| Meats, Meat/Poultry/Fish (oz/day) | 3.59 [2.27-5.48] | 4.15 [2.67-6.19] | 3.17 [2.01-4.83] |
| Meats, Organ Meats (oz/day) | 0.00 [0.00-0.03] | 0.00 [0.00-0.04] | 0.00 [0.00-0.03] |
| Meats, Poultry (oz/day) | 1.08 [0.61-1.88] | 1.14 [0.65-1.93] | 1.03 [0.59-1.83] |
| Vegetables, Total (cups/day) | 1.91 [1.26-2.87] | 1.92 [1.27-2.87] | 1.90 [1.25-2.87] |
| ***Supplement History*** | | | |
| **Consumption of Multivitamins, With or Without Minerals** | | | |
| No | 85,548 (49.17) | 42,136 (52.59) | 43,412 (46.25) |
| Yes | 88,429 (50.83) | 37,987 (47.41) | 50,442 (53.75) |
| Missing | 4,059 (2.28) | 1,541 (1.89) | 25,189 (2.61) |
| Abbreviations: Kcal, kilocalories; oz, ounces; max, maximum; min, minimum; N, number; tsp, teaspoon | | | |

| **Supplemental Table 4.** Cancer incidence in males and females, proportion of the sex differences explained by cancer risk factors using the Peters-Belson method | | | | | | | |
| --- | --- | --- | --- | --- | --- | --- | --- |
| **Cancer Site** | **Race/ethnicity** | **Incidence in men per 100,000**  **(M_inc_)** | **Incidence in women**  **per 100,000**  **(F_inc_)** | **Counterfactual per 100,000**  **(C_inc_)** | **Proportion Explained** | **Lower**  **95% CI^a^** | **Upper**  **95% CI^a^** |
| Oral cavity | African American | 149 | 76 | 97 | 71.2 | -672.2 | 814.4 |
|  | Asian American/Pacific Islander^b^ | 199 | 97 | 137 | 60.7 | -403.9 | 525.4 |
|  | Latino | 181 | 70 | 121 | 54.1 | -3.0 | 110.5 |
|  | White | 208 | 116 | 203 | 5.4 | -96.6 | 108.0 |
| Esophageal | African American | 170 | 88 | 132 | 46.3 | -171.8 | 263.4 |
| Stomach | African American | 378 | 280 | 264 | 116.3 | -1797.8 | 2029.8 |
|  | Asian American/Pacific Islander^b^ | 850 | 360 | 694 | 31.8 | 9.9 | 53.7 |
|  | Latino | 492 | 249 | 473 | 7.8 | -29.2 | 44.3 |
|  | White | 185 | 121 | 180 | 7.8 | -302.3 | 318.0 |
| Colorectal | African American | 2179 | 1653 | 2376 | -37.5 | -143.6 | 74.7 |
|  | Asian American/Pacific Islander^b^ | 2179 | 1330 | 1828 | 41.3 | 20.9 | 61.7 |
|  | Latino | 1429 | 859 | 1104 | 57.0 | 23.4 | 90.5 |
|  | White | 1357 | 966 | 1249 | 27.6 | -16.7 | 72.3 |
| Liver | African American | 363 | 113 | 313 | 20.0 | -13.6 | 53.7 |
|  | Asian American/Pacific Islander^b^ | 296 | 148 | 202 | 63.5 | 27.5 | 98.7 |
|  | Latino | 437 | 173 | 338 | 37.5 | 13.2 | 61.4 |
|  | White | 154 | 53 | 141 | 12.9 | -27.1 | 54.6 |
| Pancreas | African American | 487 | 417 | 461 | 37.1 | -1529.3 | 1602.3 |
|  | Asian American/Pacific Islander^b^ | 438 | 354 | 439 | -1.2 | -304.0 | 301.9 |
|  | Latino | 313 | 200 | 242 | 62.8 | -1363.4 | 1489.6 |
|  | White | 326 | 218 | 342 | -14.8 | -207.8 | 176.6 |
| Lung | African American | 3183 | 1903 | 2401 | 61.1 | 40.2 | 81.9 |
|  | Asian American/Pacific Islander^b^ | 1743 | 719 | 880 | 84.3 | 73.2 | 95.4 |
|  | Latino | 976 | 448 | 538 | 83.0 | 63.8 | 102.2 |
|  | White | 1731 | 1266 | 1465 | 57.2 | 6.6 | 107.7 |
| Melanoma | Latino | 118 | 60 | 110 | 13.8 | -19904.7 | 19931.9 |
|  | White | 954 | 553 | 988 | -8.5 | -18.8 | 1.8 |
| Kidney | African American | 450 | 197 | 349 | 39.9 | -4.4 | 84.0 |
|  | Asian American/Pacific Islander^b^ | 336 | 146 | 277 | 31.1 | 4.4 | 58.0 |
|  | Latino | 372 | 201 | 389 | -9.9 | -72.3 | 52.8 |
|  | White | 366 | 155 | 372 | -2.8 | -34.2 | 28.8 |
| Bladder | African American | 337 | 156 | 260 | 42.5 | -148.9 | 234.5 |
|  | Asian American/Pacific Islander^b^ | 302 | 77 | 244 | 25.9 | 12.5 | 42.4 |
|  | Latino | 272 | 54 | 220 | 23.9 | 14.5 | 55.3 |
|  | White | 272 | 54 | 220 | 23.9 | -1.3 | 22.6 |
| Brain | African American | 113 | 53 | 109 | 6.7 | -92.2 | 104.8 |
|  | Latino | 126 | 95 | 114 | 38.7 | -4522.8 | 4603.6 |
|  | White | 112 | 88 | 129 | -70.8 | -251.5 | 173.4 |
| Thyroid | African American | 59 | 69 | 80 | 210.0 | -34935.9 | 34973.2 |
|  | Asian American/Pacific Islander^b^ | 52 | 166 | 67 | 13.2 | -27.9 | 44.5 |
|  | Latino | 63 | 200 | 66 | 2.29 | -22.0 | 17.9 |
|  | White | 75 | 77 | 72 | -150.0 | -978.8 | 652.8 |
| ^a^ 95% confidence intervals were computed using normal distribution approximation for the estimated proportion explained with bootstrapped standard errors of the proportion  ^b^ Japanese American, Native Hawaiian, and other Asian American Pacific Islander individuals were combined due to small numbers in the individual groups.  Note: Not all race and ethnicity categories are shown in the table due to small cell sizes (e.g., <50 cases in each sex) due to instability of the estimates. Gallbladder cancer is not shown in this table due to small case counts by sex and race and ethnicity. | | | | | | | |
|  | | | | | | | |

| **Supplemental Table 5.** Male to female hazard ratios comparing never smokers to ever smokers for cancer sites with smoking as a risk factor. | | | | |
| --- | --- | --- | --- | --- |
| **Cancer Site** | **Age-Adjusted**  **MF HR (95% CI)**  **in Never Smokers** | **Age-Adjusted**  **MF HR (95% CI)**  **in Ever Smokers** | **Fully adjusted**  **MF HR (95% CI)**  **in Never Smokers** | **Fully adjusted**  **MF HR (95% CI)**  **in Ever Smokers***^k^* |
| Oral Cavity | 1.29 (0.90, 1.86) | 1.98 (1.57, 2.51) | 1.16 (0.76, 1.75)*^a^* | 1.77 (1.35, 2.32) |
| Esophageal | 4.22 (2.54, 7.04) | 3.54 (2.62, 4.80) | 4.53 (2.54, 8.07)*^b^* | 3.35 (2.38, 4.71) |
| Stomach | 1.60 (1.33, 1.93) | 1.82 (1.57, 2.11) | 1.68 (1.36, 2.07)*^c^* | 1.50 (1.27, 1.79) |
| Colorectal | 1.29 (1.17, 1.42) | 1.37 (1.27, 1.47) | 1.31 (1.18, 1.45)*^d^* | 1.22 (1.12, 1.33) |
| Liver | 1.55 (1.22, 1.98) | 2.33 (1.94, 2.79) | 1.39 (1.07, 1.81)*^e^* | 2.09 (1.71, 2.56) |
| Gallbladder | 0.38 (0.20, 0.74) | 0.90 (0.57, 1.42) | 0.35 (0.17, 0.69)*^f^* | 0.87 (0.52, 1.46) |
| Pancreas | 1.09 (0.93, 1.29) | 1.13 (0.99, 1.30) | 1.16 (0.97, 1.40)*^g^* | 1.12 (0.96, 1.32) |
| Lung | 1.00 (0.83, 1.20) | 1.08 (1.02, 1.15) | 1.01 (0.83, 1.22)*^h^* | 1.11 (1.04, 1.19) |
| Kidney | 1.77 (1.46, 2.16) | 1.80 (1.52, 2.12) | 1.83 (1.48, 2.28)*^i^* | 1.82 (1.50, 2.21) |
| Bladder | 3.23 (2.45, 4.27) | 2.93 (2.44, 3.52) | 3.25 (2.43, 4.34)*^j^* | 3.00 (2.47, 3.65) |
| Abbreviations: CI, confidence interval; MF HR, male-to-female hazard ratio  *^a^* Adjusted for age (continuous), occupation (laborer/farm worker/factory worker/machine operator, clerical/office worker/sales/manager/administrator, craftsperson/small business owner/professional/technical, unemployed/other occupation or missing), BMI (normal, underweight, overweight, obese), alcohol use (no drinking, low to moderate drinking, heavy drinking), physical activity (sedentary, low to moderate activity, vigorous activity), calorie intake (25^th^,50^th^, 75^th^ and 100^th^ quartile, kcal/day), sugar intake (25th,50th, 75th and 100th quartile, tsp/day), dairy intake (25th,50th, 75th and 100th quartile, cups/day), total fruit intake (25th,50th, 75th and 100th quartile, cups/day), total fruit juice intake (25th,50th, 75th and 100th quartile, cups/day), total grains intake (25th,50th, 75th and 100th quartile, cups/day), all meat intake-including poultry and fish (25th,50th, 75th and 100th quartile), ounces/day), total vegetable intake (25th,50th, 75th and 100th quartile, cups/day), fish with low omega-3 (25th,50th, 75th and 100th quartile, ounces/day), fish with high omega-3 (25th,50th, 75th and 100th quartile, ounces/day), processed meat intake (25^th^,50^th^, 75^th^ and 100^th^ quartile, ounces/day) and meat intake (25^th^,50^th^, 75^th^ and 100^th^ quartile, ounces/day).  *^b^* Adjusted for age (continuous), BMI (normal, underweight, overweight, obese), smoking status (never smoker, past, current), smoking duration and intensity (never smoker, light smoker, moderate smoker, heavy smoker), physical activity (sedentary, low to moderate activity, vigorous activity), alcohol use (no drinking, low to moderate drinking, heavy drinking), antacid use (no, yes-but not currently, yes-currently), and Tagamet, Zantac, or Pepcid use (no, yes-but not currently, yes-currently).  *^c^* Adjusted for age (continuous), race/ethnicity (African American, Japanese American, Native Hawaiian , Latino, White), place of birth (US born or foreign born), occupation (laborer/farm worker/factory worker/machine operator, clerical/office worker/sales/manager/administrator, craftsperson/small business owner/professional/technical, unemployed/other occupation or missing), BMI (normal, underweight, overweight, obese), history of polyps of intestines (yes or no), history of partial removal of the stomach (yes or no), family history of stomach cancer (yes or no), history of high blood pressure (yes or no), total fruit intake (25th,50th, 75th and 100th quartile, cups/day), total fruit juice intake (25th,50th, 75th and 100th quartile, cups/day), total grain intake (25th,50th, 75th and 100th quartile, cups/day), all meat intake-including poultry and fish (25th,50th, 75th and 100th quartile), total vegetable intake (25th,50th, 75th and 100th quartile, cups/day), fish with low omega-3 (25th,50th, 75th and 100th quartile, ounces/day), fish with high omega-3 (25th,50th, 75th and 100th quartile, ounces/day), processed meat intake (25^th^,50^th^, 75^th^ and 100^th^ quartile, ounces/day) and meat intake (25^th^,50^th^, 75^th^ and 100^th^ quartile, ounces/day), alcohol use (no drinking, low to moderate drinking, heavy drinking), smoking status (never smoker, past, current) smoking duration and intensity (never smoker, light smoker, moderate smoker, heavy smoker), and history of ulcers (yes or no).  *^d^* Adjusted for age (continuous), history of polyps of intestines (yes or no), race/ethnicity (African American, Japanese American, Native Hawaiian , Latino, White), BMI (normal, underweight, overweight, obese), physical activity (sedentary, low to moderate activity, vigorous activity), calorie intake (25^th^,50^th^, 75^th^ and 100^th^ quartile, kcal/day), alcohol use (no drinking, low to moderate drinking, heavy drinking), sugar intake (25th,50th, 75th and 100th quartile, tsp/day), smoking status (never smoker, past, current), smoking duration and intensity (never smoker, light smoker, moderate smoker, heavy smoker), processed meat intake (25^th^,50^th^, 75^th^ and 100^th^ quartile, ounces/day), meat intake (25^th^,50^th^, 75^th^ and 100^th^ quartile, ounces/day), organ meat intake (less than 1 ounce/day or 1+ ounces/day) and consumption of multivitamins (yes or no).  *^e^* Adjusted for age (continuous), history of diabetes (yes or no), BMI (normal, underweight, overweight, obese), alcohol use (no drinking, low to moderate drinking, heavy drinking), sugar intake (25th,50th, 75th and 100th quartile, tsp/day), race/ethnicity (African American, Japanese American, Native Hawaiian , Latino, White) and smoking status (never smoker, past, current) and smoking duration and intensity (never smoker, light smoker, moderate smoker, heavy smoker).  *^f^* Adjusted for age (continuous), history of gallstones (yes or no), race/ethnicity (African American, Japanese American, Native Hawaiian , Latino, White), occupation (laborer/farm worker/factory worker/machine operator, clerical/office worker/sales/manager/administrator, craftsperson/small business owner/professional/technical, unemployed/other occupation or missing), BMI (normal, underweight, overweight, obese), and smoking status (never smoker, past, current), and smoking duration and intensity (never smoker, light smoker, moderate smoker, heavy smoker).  *^g^* Adjusted for age (continuous), occupation (laborer/farm worker/factory worker/machine operator, clerical/office worker/sales/manager/administrator, craftsperson/small business owner/professional/technical, unemployed/other occupation or missing), smoking status (never smoker, past, current), smoking duration and intensity (never smoker, light smoker, moderate smoker, heavy smoker), alcohol use (no drinking, low to moderate drinking, heavy drinking), physical activity (sedentary, low to moderate activity, vigorous activity), ), calorie intake (25^th^,50^th^, 75^th^ and 100^th^ quartile, kcal/day), sugar intake (25th,50th, 75th and 100th quartile, tsp/day), history of diabetes (yes or no), processed meat intake (25^th^,50^th^, 75^th^ and 100^th^ quartile, ounces/day), meat intake (25^th^,50^th^, 75^th^ and 100^th^ quartile, ounces/day), family history of pancreatic cancer (yes or no) and race/ethnicity (African American, Japanese American, Native Hawaiian , Latino, White).  *^h^* Adjusted for age (continuous), family history of lung cancer (yes or no), place of birth (US born or foreign born), smoking status (never smoker, past, current) smoking duration and intensity (never smoker, light smoker, moderate smoker, heavy smoker), and occupation (laborer/farm worker/factory worker/machine operator, clerical/office worker/sales/manager/administrator, craftsperson/small business owner/professional/technical, unemployed/other occupation or missing).  *^i^* Adjusted for age (continuous), family history of kidney cancer (yes or no), BMI (normal, underweight, overweight, obese), smoking status (never smoker, past, current), smoking duration and intensity (never smoker, light smoker, moderate smoker, heavy smoker), occupation (laborer/farm worker/factory worker/machine operator, clerical/office worker/sales/manager/administrator, craftsperson/small business owner/professional/technical, unemployed/other occupation or missing), history of high blood pressure (yes or no), acetaminophen use (no, yes-but not currently, yes-currently), aspirin use (no, yes-but not currently, yes-currently), and race/ethnicity (African American, Japanese American, Native Hawaiian , Latino, White).  *^j^* Adjusted for age (continuous), race/ethnicity (African American, Japanese American, Native Hawaiian , Latino, White), place of birth (US born or foreign born), smoking status (never smoker, past, current), smoking duration and intensity (never smoker, light smoker, moderate smoker, heavy smoker), history of diabetes (yes or no) and occupation (laborer/farm worker/factory worker/machine operator, clerical/office worker/sales/manager/administrator, craftsperson/small business owner/professional/technical, unemployed/other occupation or missing).  *^K^* Adjusted for smoking packyears (light smoker, moderate smoker, heavy smoker) in addition to the same risk factors as corresponding fully adjusted model in never smokers. | | | | |

| **Supplemental Table 6.** Fully adjusted male-to-female hazard ratios among never smokers by race and ethnicity. | | | | | | |
| --- | --- | --- | --- | --- | --- | --- |
| **Cancer Site** | **African American** | **Japanese American** | **Native Hawaiian** | **Latino** | **White** | **Interaction**  ***P*-values** *^a^* |
|  | **MF HR (95% CI)** | **MF HR (95% CI)** | **MF HR (95% CI)** | **MF HR (95% CI)** | **MF HR (95% CI)** |  |
| Oral Cavity *^b^* | 0.21 (0.02, 1.92) | 1.14 (0.61, 2.12) | 5.53 (0.99, 30.93) | 0.98 (0.35, 2.75) | 1.47 (0.59, 3.70) | 0.448 |
| Esophageal *^c^* | 4.25 (1.14, 15.90) | 5.29 (1.62, 17.23) | NE | 2.71 (0.84, 8.74) | 4.66 (1.62, 13.38) | 0.895 |
| Stomach *^d^* | 1.75 (0.90, 3.40) | 1.79 (1.31, 2.47) | 1.58 (0.66, 3.76) | 1.45 (0.97, 2.16) | 2.47 (1.20, 5.11) | 0.707 |
| Colorectal *^e^* | 1.55 (1.21, 1.98) | 1.26 (1.06, 1.49) | 1.23 (0.80, 1.91) | 1.38 (1.10, 1.73) | 1.16 (0.91, 1.48) | 0.402 |
| Liver *^f^* | 2.95 (1.26, 6.92) | 1.25 (0.79, 1.96) | 1.49 (0.47, 4.70) | 1.22 (0.81, 1.85) | 1.31 (0.62, 2.81) | 0.419 |
| Gallbladder *^g^* | 0.90 (0.10, 8.14) | 0.41 (0.05, 3.44) | NE | 0.35 (0.14, 0.86) | 0.36 (0.08, 1.69) | 0.954 |
| Pancreas *^h^* | 0.98 (0.60, 1.59) | 1.28 (0.94, 1.75) | 1.21 (0.65, 2.23) | 1.09 (0.74, 1.61) | 1.16 (0.77, 1.77) | 0.872 |
| Lung *^i^* | 1.22 (0.74, 2.02) | 1.23 (0.93, 1.69) | 0.66 (0.30, 1.44) | 0.81 (0.54, 1.20) | 0.94 (0.60, 1.45) | 0.356 |
| Kidney *^j^* | 1.25 (0.59, 2.67) | 1.57 (1.04, 2.36) | 1.62 (0.84, 3.12) | 1.75 (1.24, 2.47) | 3.24 (1.88, 5.58) | 0.295 |
| Bladder *^k^* | 2.01 (0.89, 4.52) | 3.16 (1.95, 5.12) | 4.36 (1.42, 13.44) | 6.04 (3.10, 11.76) | 2.24 (1.18, 4.25) | 0.144 |
| Abbreviations: CI, confidence interval; MF HR, male-to-female hazard ratio; NE, not estimable  *^a^ P-*value from Wald test assessing the statistical significance of the estimated interaction term for sex by race/ethnicity in the Cox proportional hazards model.  *^b^* Adjusted for age (continuous), occupation (laborer/farm worker/factory worker/machine operator, clerical/office worker/sales/manager/administrator, craftsperson/small business owner/professional/technical, unemployed/other occupation or missing), BMI (normal, underweight, overweight, obese), alcohol use (no drinking, low to moderate drinking, heavy drinking), physical activity (sedentary, low to moderate activity, vigorous activity), calorie intake (25^th^,50^th^, 75^th^ and 100^th^ quartile, kcal/day), sugar intake (25th,50th, 75th and 100th quartile, tsp/day), dairy intake (25th,50th, 75th and 100th quartile, cups/day), total fruit intake (25th,50th, 75th and 100th quartile, cups/day), total fruit juice intake (25th,50th, 75th and 100th quartile, cups/day), total grains intake (25th,50th, 75th and 100th quartile, cups/day), all meat intake-including poultry and fish (25th,50th, 75th and 100th quartile), ounces/day), total vegetable intake (25th,50th, 75th and 100th quartile, cups/day), fish with low omega-3 (25th,50th, 75th and 100th quartile, ounces/day), fish with high omega-3 (25th,50th, 75th and 100th quartile, ounces/day), processed meat intake (25^th^,50^th^, 75^th^ and 100^th^ quartile, ounces/day) and meat intake (25^th^,50^th^, 75^th^ and 100^th^ quartile, ounces/day).  *^c^* Adjusted for age (continuous), BMI (normal, underweight, overweight, obese), physical activity (sedentary, low to moderate activity, vigorous activity), alcohol use (no drinking, low to moderate drinking, heavy drinking), antacid use (no, yes-but not currently, yes-currently), and Tagamet, Zantac, or Pepcid use (no, yes-but not currently, yes-currently).  *^d^* Adjusted for age (continuous), place of birth (US born or foreign born), occupation (laborer/farm worker/factory worker/machine operator, clerical/office worker/sales/manager/administrator, craftsperson/small business owner/professional/technical, unemployed/other occupation or missing), BMI (normal, underweight, overweight, obese), history of polyps of intestines (yes or no), history of partial removal of the stomach (yes or no), family history of stomach cancer (yes or no), history of high blood pressure (yes or no), total fruit intake (25th,50th, 75th and 100th quartile, cups/day), total fruit juice intake (25th,50th, 75th and 100th quartile, cups/day), total grain intake (25th,50th, 75th and 100th quartile, cups/day), all meat intake-including poultry and fish (25th,50th, 75th and 100th quartile), total vegetable intake (25th,50th, 75th and 100th quartile, cups/day), fish with low omega-3 (25th,50th, 75th and 100th quartile, ounces/day), fish with high omega-3 (25th,50th, 75th and 100th quartile, ounces/day), processed meat intake (25^th^,50^th^, 75^th^ and 100^th^ quartile, ounces/day) and meat intake (25^th^,50^th^, 75^th^ and 100^th^ quartile, ounces/day), alcohol use (no drinking, low to moderate drinking, heavy drinking), smoking status (never smoker, past, current) , and history of ulcers (yes or no).  *^e^* Adjusted for age (continuous), history of polyps of intestines (yes or no), , BMI (normal, underweight, overweight, obese), physical activity (sedentary, low to moderate activity, vigorous activity), calorie intake (25^th^,50^th^, 75^th^ and 100^th^ quartile, kcal/day), alcohol use (no drinking, low to moderate drinking, heavy drinking), sugar intake (25th,50th, 75th and 100th quartile, tsp/day), , processed meat intake (25^th^,50^th^, 75^th^ and 100^th^ quartile, ounces/day), meat intake (25^th^,50^th^, 75^th^ and 100^th^ quartile, ounces/day), organ meat intake (less than 1 ounce/day or 1+ ounces/day) and consumption of multivitamins (yes or no).  *^f^* Adjusted for age (continuous), history of diabetes (yes or no), BMI (normal, underweight, overweight, obese), alcohol use (no drinking, low to moderate drinking, heavy drinking), and sugar intake (25th,50th, 75th and 100th quartile, tsp/day).  *^g^* Adjusted for age (continuous), history of gallstones (yes or no), occupation (laborer/farm worker/factory worker/machine operator, clerical/office worker/sales/manager/administrator, craftsperson/small business owner/professional/technical, unemployed/other occupation or missing), BMI (normal, underweight, overweight, obese), and and .  *^h^* Adjusted for age (continuous), occupation (laborer/farm worker/factory worker/machine operator, clerical/office worker/sales/manager/administrator, craftsperson/small business owner/professional/technical, unemployed/other occupation or missing), , alcohol use (no drinking, low to moderate drinking, heavy drinking), physical activity (sedentary, low to moderate activity, vigorous activity), calorie intake (25^th^,50^th^, 75^th^ and 100^th^ quartile, kcal/day), sugar intake (25th,50th, 75th and 100th quartile, tsp/day), history of diabetes (yes or no), processed meat intake (25^th^,50^th^, 75^th^ and 100^th^ quartile, ounces/day), meat intake (25^th^,50^th^, 75^th^ and 100^th^ quartile, ounces/day), family history of pancreatic cancer (yes or no) and.  *^i^* Adjusted for age (continuous), family history of lung cancer (yes or no), place of birth (US born or foreign born), and occupation (laborer/farm worker/factory worker/machine operator, clerical/office worker/sales/manager/administrator, craftsperson/small business owner/professional/technical, unemployed/other occupation or missing).  *^j^* Adjusted for age (continuous), family history of kidney cancer (yes or no), BMI (normal, underweight, overweight, obese), occupation (laborer/farm worker/factory worker/machine operator, clerical/office worker/sales/manager/administrator, craftsperson/small business owner/professional/technical, unemployed/other occupation or missing), history of high blood pressure (yes or no), acetaminophen use (no, yes-but not currently, yes-currently), aspirin use (no, yes-but not currently, yes-currently.  *^k^* Adjusted for age (continuous), place of birth (US born or foreign born), history of diabetes (yes or no) and occupation (laborer/farm worker/factory worker/machine operator, clerical/office worker/sales/manager/administrator, craftsperson/small business owner/professional/technical, unemployed/other occupation or missing). | | | | | | |

| **Supplemental Table 7.** Fully adjusted male-to-female hazard ratios among ever smokers by race/ethnicity. | | | | | | |
| --- | --- | --- | --- | --- | --- | --- |
| **Cancer Site** | **African American** | **Japanese American** | **Native Hawaiian** | **Latino** | **White** | **Interaction**  ***P*-values** *^a^* |
|  | **MF HR (95% CI)** | **MF HR (95% CI)** | **MF HR (95% CI)** | **MF HR (95% CI)** | **MF HR (95% CI)** |  |
| Oral Cavity *^b^* | 1.24 (0.63, 2.45) | 1.27 (0.70, 2.32) | 1.94 (0.65, 5.74) | 2.27 (1.23, 4.22) | 2.06 (1.30, 3.26) | 0.973 |
| Esophageal *^c^* | 0.99 (0.51, 1.92) | 5.46 (1.93, 15.41) | 14.44 (1.77, 117.62) | 7.94 (2.82, 22.39) | 3.10 (1.70, 5.63) | 0.008 |
| Stomach *^d^* | 1.07 (0.71, 1.61) | 1.67 (1.24, 2.24) | 2.46 (1.31, 4.63) | 1.48 (1.03, 2.12) | 1.36 (0.83, 2.23) | 0.100 |
| Colorectal *^e^* | 1.10 (0.91, 1.33) | 1.36 (1.15, 1.61) | 1.22 (0.89, 1.67) | 1.18 (0.96, 1.45) | 1.20 (1.00, 1.45) | 0.311 |
| Liver *^f^* | 2.42 (1.52, 3.85) | 1.31 (0.96, 1.91) | 2.88 (1.38, 6.04) | 1.75 (1.24, 2.49) | 4.04 (2.30, 7.11) | 0.036 |
| Gallbladder *^g^* | 0.59 (0.18, 1.94) | 1.45 (0.48, 4.43) | NE | 0.34 (0.14, 0.82) | 1.49 (0.39, 5.73) | 0.528 |
| Pancreas *^h^* | 0.98 (0.70, 1.38) | 0.95 (0.71, 1.27) | 1.21 (0.72, 2.04) | 1.36 (0.91, 2.04) | 1.37 (0.96, 1.95) | 0.274 |
| Lung *^i^* | 1.15 (1.01, 1.31) | 1.17 (1.01, 1.35) | 1.31 (1.08, 1.60) | 1.10 (0.91, 1.33) | 0.98 (0.87, 1.11) | 0.320 |
| Kidney *^j^* | 1.70 (1.12, 2.58) | 1.44 (0.97, 2.13) | 3.20 (1.68, 6.13) | 1.77 (1.19, 2.63) | 1.78 (1.23, 2.57) | 0.502 |
| Bladder *^k^* | 2.57 (1.69, 3.91) | 2.93 (1.85, 4.65) | 1.98 (1.10, 3.89) | 2.86 (1.69, 4.86) | 3.77 (2.72, 5.24) | 0.328 |
| Abbreviations: CI, confidence interval; MF HR, male-to-female hazard ratio; NE, not estimable  *^a^ P-*value from Wald test assessing the statistical significance of the estimated interaction term for sex by race/ethnicity in the Cox proportional hazards model.  *^b^* Adjusted for age (continuous), occupation (laborer/farm worker/factory worker/machine operator, clerical/office worker/sales/manager/administrator, craftsperson/small business owner/professional/technical, unemployed/other occupation or missing), BMI (normal, underweight, overweight, obese), smoking packyears (light smoker, moderate smoker, heavy smoker), alcohol use (no drinking, low to moderate drinking, heavy drinking), physical activity (sedentary, low to moderate activity, vigorous activity), calorie intake (25^th^,50^th^, 75^th^ and 100^th^ quartile, kcal/day), sugar intake (25th,50th, 75th and 100th quartile, tsp/day), dairy intake (25th,50th, 75th and 100th quartile, cups/day), total fruit intake (25th,50th, 75th and 100th quartile, cups/day), total fruit juice intake (25th,50th, 75th and 100th quartile, cups/day), total grains intake (25th,50th, 75th and 100th quartile, cups/day), all meat intake-including poultry and fish (25th,50th, 75th and 100th quartile), ounces/day), total vegetable intake (25th,50th, 75th and 100th quartile, cups/day), fish with low omega-3 (25th,50th, 75th and 100th quartile, ounces/day), fish with high omega-3 (25th,50th, 75th and 100th quartile, ounces/day), processed meat intake (25^th^,50^th^, 75^th^ and 100^th^ quartile, ounces/day) and meat intake (25^th^,50^th^, 75^th^ and 100^th^ quartile, ounces/day).  *^c^* Adjusted for age (continuous), BMI (normal, underweight, overweight, obese), smoking packyears (light smoker, moderate smoker, heavy smoker), physical activity (sedentary, low to moderate activity, vigorous activity), alcohol use (no drinking, low to moderate drinking, heavy drinking), antacid use (no, yes-but not currently, yes-currently), and Tagamet, Zantac, or Pepcid use (no, yes-but not currently, yes-currently).  *^d^* Adjusted for age (continuous), place of birth (US born or foreign born), occupation (laborer/farm worker/factory worker/machine operator, clerical/office worker/sales/manager/administrator, craftsperson/small business owner/professional/technical, unemployed/other occupation or missing), BMI (normal, underweight, overweight, obese), history of polyps of intestines (yes or no), history of partial removal of the stomach (yes or no), family history of stomach cancer (yes or no), history of high blood pressure (yes or no), total fruit intake (25th,50th, 75th and 100th quartile, cups/day), total fruit juice intake (25th,50th, 75th and 100th quartile, cups/day), total grain intake (25th,50th, 75th and 100th quartile, cups/day), all meat intake-including poultry and fish (25th,50th, 75th and 100th quartile), total vegetable intake (25th,50th, 75th and 100th quartile, cups/day), fish with low omega-3 (25th,50th, 75th and 100th quartile, ounces/day), fish with high omega-3 (25th,50th, 75th and 100th quartile, ounces/day), processed meat intake (25^th^,50^th^, 75^th^ and 100^th^ quartile, ounces/day) and meat intake (25^th^,50^th^, 75^th^ and 100^th^ quartile, ounces/day), alcohol use (no drinking, low to moderate drinking, heavy drinking), smoking packyears (light smoker, moderate smoker, heavy smoker), and history of ulcers (yes or no).  *^e^* Adjusted for age (continuous), history of polyps of intestines (yes or no), BMI (normal, underweight, overweight, obese), physical activity (sedentary, low to moderate activity, vigorous activity), calorie intake (25^th^,50^th^, 75^th^ and 100^th^ quartile, kcal/day), alcohol use (no drinking, low to moderate drinking, heavy drinking), sugar intake (25th,50th, 75th and 100th quartile, tsp/day), smoking packyears (light smoker, moderate smoker, heavy smoker), processed meat intake (25^th^,50^th^, 75^th^ and 100^th^ quartile, ounces/day), meat intake (25^th^,50^th^, 75^th^ and 100^th^ quartile, ounces/day), organ meat intake (less than 1 ounce/day or 1+ ounces/day) and consumption of multivitamins (yes or no).  *^f^* Adjusted for age (continuous), history of diabetes (yes or no), BMI (normal, underweight, overweight, obese), alcohol use (no drinking, low to moderate drinking, heavy drinking), sugar intake (25th,50th, 75th and 100th quartile, tsp/day), smoking packyears (light smoker, moderate smoker, heavy smoker).  *^g^* Adjusted for age (continuous), history of gallstones (yes or no), occupation (laborer/farm worker/factory worker/machine operator, clerical/office worker/sales/manager/administrator, craftsperson/small business owner/professional/technical, unemployed/other occupation or missing), BMI (normal, underweight, overweight, obese), and smoking packyears (light smoker, moderate smoker, heavy smoker).  *^h^* Adjusted for age (continuous), occupation (laborer/farm worker/factory worker/machine operator, clerical/office worker/sales/manager/administrator, craftsperson/small business owner/professional/technical, unemployed/other occupation or missing), smoking packyears (light smoker, moderate smoker, heavy smoker), alcohol use (no drinking, low to moderate drinking, heavy drinking), physical activity (sedentary, low to moderate activity, vigorous activity), calorie intake (25^th^,50^th^, 75^th^ and 100^th^ quartile, kcal/day), sugar intake (25th,50th, 75th and 100th quartile, tsp/day), history of diabetes (yes or no), processed meat intake (25^th^,50^th^, 75^th^ and 100^th^ quartile, ounces/day), meat intake (25^th^,50^th^, 75^th^ and 100^th^ quartile, ounces/day), family history of pancreatic cancer (yes or no).  *^i^* Adjusted for age (continuous), family history of lung cancer (yes or no), place of birth (US born or foreign born), smoking packyears (light smoker, moderate smoker, heavy smoker), and occupation (laborer/farm worker/factory worker/machine operator, clerical/office worker/sales/manager/administrator, craftsperson/small business owner/professional/technical, unemployed/other occupation or missing).  *^j^* Adjusted for age (continuous), family history of kidney cancer (yes or no), BMI (normal, underweight, overweight, obese), smoking packyears (light smoker, moderate smoker, heavy smoker), occupation (laborer/farm worker/factory worker/machine operator, clerical/office worker/sales/manager/administrator, craftsperson/small business owner/professional/technical, unemployed/other occupation or missing), history of high blood pressure (yes or no), acetaminophen use (no, yes-but not currently, yes-currently), aspirin use (no, yes-but not currently, yes-currently).  *^k^* Adjusted for age (continuous), place of birth (US born or foreign born), smoking packyears (light smoker, moderate smoker, heavy smoker), history of diabetes (yes or no) and occupation (laborer/farm worker/factory worker/machine operator, clerical/office worker/sales/manager/administrator, craftsperson/small business owner/professional/technical, unemployed/other occupation or missing). | | | | | | |

| **Supplemental Table 8.** Probabilistic sensitivity analyses of the effects of unmeasured differences between men and women in exposure to hepatitis B and C on liver cancer | | | | | |
| --- | --- | --- | --- | --- | --- |
| **Race/ethnicity** | **Prevalence of infectious agent among men** | **Prevalence of infectious agent among women** | **RR range for infectious agent and cancer site^b^** | **MF HR_crude_ for Liver Cancer** | **MF HR_bias_ _adjusted_ for Liver Cancer** |
|  |  |  |  |  |  |
| **Chronic hepatitis B virus ^a^** | | | | | |
| Black | 22.89 | 19.24 | 2.2–11.0 | 3.05 | 2.20 |
| Hispanic | 10.12 | 4.77 | 0.4–15.5 | 2.04 | 1.74 |
| Asian | 28.37 | 24.34 | 23.2–42.2 | 1.93 | 1.81 |
| White | 5.87 | 2.86 | 10.5–19.8 | 2.87 | 2.36 |
| **Chronic hepatitis C virus ^a^** | | | | | |
| Black | 5.79 | 3.22 | 51.3–79.3 | 3.05 | 2.49 |
| Hispanic | 4.10 | 2.26 | 26.9–59.9 | 2.04 | 1.85 |
| Asian | 4.16 | 2.67 | 44.4–75.2 | 1.93 | 2.39 |
| White | 1.87 | 0.86 | 54.9–67.5 | 2.87 | 2.51 |
| Abbreviations: NHANES, National Health and Nutrition Examination Survey; RR, relative risk  ^a^ Prevalence estimated from NHANES III in adults 50–70. Only individuals positive for hepatitis B core antibody were tested for hepatitis B surface antigen. Individuals positive for hepatitis C antibodies received a nucleic acid test for HCV RNA.  ^b^ Makarova-Rusher, OV. et al. Population Attributable Fractions of Risk Factors for Hepatocellular Carcinoma in the United States. *Cancer*. 2016; 122(11): 1757–1765. | | | | | |

**Reference**

1. Bladder Cancer Risk Factors. American Cancer Society. Updated March 12, 2024. Accessed September 6, 2024. <https://www.cancer.org/cancer/types/bladder-cancer/causes-risks-prevention/risk-factors.html>

2. Cancer Stat Facts: Bladder Cancer. Surveillance, Epidemiology, and End Results (SEER); National Cancer Institute 2024. <https://seer.cancer.gov/statfacts/html/urinb.html>

3. Cumberbatch MGK, Jubber I, Black PC, et al. Epidemiology of Bladder Cancer: A Systematic Review and Contemporary Update of Risk Factors in 2018. *Eur Urol*. Dec 2018;74(6):784-795. doi:10.1016/j.eururo.2018.09.001

4. Wang Y, Chang Q, Li Y. Racial differences in Urinary Bladder Cancer in the United States. *Sci Rep*. Aug 21 2018;8(1):12521. doi:10.1038/s41598-018-29987-2

5. Wong MCS, Fung FDH, Leung C, Cheung WWL, Goggins WB, Ng CF. The global epidemiology of bladder cancer: a joinpoint regression analysis of its incidence and mortality trends and projection. *Sci Rep*. Jan 18 2018;8(1):1129. doi:10.1038/s41598-018-19199-z

6. Yan H, Xie H, Ying Y, Li J, Wang X, Xu X, Zheng X. Pioglitazone use in patients with diabetes and risk of bladder cancer: a systematic review and meta-analysis. *Cancer Manag Res*. 2018;10:1627-1638. doi:10.2147/CMAR.S164840

7. Risk Factors for Gallbladder Cancer. American Cancer Society. Updated 2018. Accessed September 6, 2024. <https://www.cancer.org/cancer/types/gallbladder-cancer/causes-risks-prevention/risk-factors.html>

8. Perez-Moreno P, Riquelme I, Garcia P, Brebi P, Roa JC. Environmental and Lifestyle Risk Factors in the Carcinogenesis of Gallbladder Cancer. *J Pers Med*. Feb 8 2022;12(2)doi:10.3390/jpm12020234

9. Renehan AG, Tyson M, Egger M, Heller RF, Zwahlen M. Body-mass index and incidence of cancer: a systematic review and meta-analysis of prospective observational studies. *Lancet*. Feb 16 2008;371(9612):569-78. doi:10.1016/S0140-6736(08)60269-X

10. Wenbin D, Zhuo C, Zhibing M, et al. The effect of smoking on the risk of gallbladder cancer: a meta-analysis of observational studies. *Eur J Gastroenterol Hepatol*. Mar 2013;25(3):373-9. doi:10.1097/MEG.0b013e32835a870b

11. Risk Factors for Oral Cavity and Oropharyngeal Cancers. American Cancer Society. Updated 2021. Accessed September 6, 2024. <https://www.cancer.org/cancer/types/oral-cavity-and-oropharyngeal-cancer/causes-risks-prevention/risk-factors.html>

12. Lung cancer risk factors. American Cancer Society. Updated 2024. Accessed September 6, 2024. <https://www.cancer.org/cancer/types/lung-cancer/causes-risks-prevention/risk-factors.html>

13. Lung cancer: Lung cancer risk factors. National Center for Chronic Disease Prevention and Health Promotion. Updated 2023. Accessed September 6, 2024. <https://www.cdc.gov/lung-cancer/risk-factors/index.html>

14. Pallis AG, Syrigos KN. Lung cancer in never smokers: disease characteristics and risk factors. *Crit Rev Oncol Hematol*. Dec 2013;88(3):494-503. doi:10.1016/j.critrevonc.2013.06.011

15. Risk factors for brain and spinal cord tumors. American Cancer Society. Updated May 5, 2020. Accessed September 06, 2024. <https://www.cancer.org/cancer/types/brain-spinal-cord-tumors-adults/causes-risks-prevention/risk-factors.html>

16. Michaud D. and Batchelor T. Risk factors for brain tumors. UpToDate. Updated February 7, 2020. Accessed September 6, 2024. <https://www.uptodate.com/contents/risk-factors-for-brain-tumors>

17. Risk Factors for Kidney Cancer. American Cancer Society. Updated 2024. Accessed September 6, 2024. <https://www.cancer.org/cancer/types/kidney-cancer/causes-risks-prevention/risk-factors.html>

18. Gelfond J, Al-Bayati O, Kabra A, Iffrig K, Kaushik D, Liss MA. Modifiable risk factors to reduce renal cell carcinoma incidence: Insight from the PLCO trial. *Urol Oncol*. Jul 2018;36(7):340 e1-340 e6. doi:10.1016/j.urolonc.2018.04.011

19. Kabaria R, Klaassen Z, Terris MK. Renal cell carcinoma: links and risks. *Int J Nephrol Renovasc Dis*. 2016;9:45-52. doi:10.2147/IJNRD.S75916

20. Macleod LC, Hotaling JM, Wright JL, Davenport MT, Gore JL, Harper J, White E. Risk factors for renal cell carcinoma in the VITAL study. *J Urol*. Nov 2013;190(5):1657-61. doi:10.1016/j.juro.2013.04.130

21. Pancreatic cancer risk factors. American Cancer Society. Updated 2024. Accessed September 6, 2024. <https://www.cancer.org/cancer/types/pancreatic-cancer/causes-risks-prevention/risk-factors.html>

22. Colorectal Cancer Risk Factors. American Cancer Society. Accessed September 06, 2024. <https://www.cancer.org/cancer/types/colon-rectal-cancer/causes-risks-prevention/risk-factors.html>

23. Genetics of Colorectal Cancer (PDQ®)–Health Professional Version. National Cancer Institute. Updated 2023. Accessed September 6, 2024. <https://www.cancer.gov/types/colorectal/hp/colorectal-genetics-pdq#_2701_toc>

24. Keum N, Giovannucci E. Global burden of colorectal cancer: emerging trends, risk factors and prevention strategies. *Nat Rev Gastroenterol Hepatol*. Dec 2019;16(12):713-732. doi:10.1038/s41575-019-0189-8

25. Rock CL, Thomson C, Gansler T, et al. American Cancer Society guideline for diet and physical activity for cancer prevention. *CA Cancer J Clin*. Jul 2020;70(4):245-271. doi:10.3322/caac.21591

26. Liver Cancer Risk Factors. American Cancer Society. Updated 2019. Accessed September 06, 2024. <https://www.cancer.org/cancer/types/liver-cancer/causes-risks-prevention/risk-factors.html>

27. Asafo-Agyei KO, Samant H. Hepatocellular Carcinoma. *StatPearls*. 2024.

28. Chidambaranathan-Reghupaty S, Fisher PB, Sarkar D. Hepatocellular carcinoma (HCC): Epidemiology, etiology and molecular classification. *Adv Cancer Res*. 2021;149:1-61. doi:10.1016/bs.acr.2020.10.001

29. Stomach cancer risk factors. American Cancer Society. Updated 2021. Accessed September 6, 2024. <https://www.cancer.org/cancer/types/stomach-cancer/causes-risks-prevention/risk-factors.html>

30. Esophageal Cancer Risk Factors. American Cancer Society. Updated 2020. Accessed September 6, 2024. <https://www.cancer.org/cancer/types/esophagus-cancer/causes-risks-prevention/risk-factors.html>

31. Zhang Y. Epidemiology of esophageal cancer. *World J Gastroenterol*. Sep 14 2013;19(34):5598-606. doi:10.3748/wjg.v19.i34.5598

32. Risk Factors for Melanoma Skin Cancer. American Cancer Society. Updated 2023. Accessed September 6, 2024. <https://www.cancer.org/cancer/types/melanoma-skin-cancer/causes-risks-prevention/risk-factors.html>

33. SEER Cancer Stat Facts: Melanoma of the Skin. Updated 2023. Accessed September 6, 2024. <https://seer.cancer.gov/statfacts/html/melan.html>

34. Curiel-Lewandrowski C. Melanoma: Epidemiology and risk factors. UpToDate. Updated 2023. Accessed Septmeber 15, 2024. <https://www.uptodate.com/contents/melanoma-epidemiology-and-risk-factors>

35. Thyroid cancer risk factors. American Cancer Society. Updated 2024. Accessed September 6, 2024. <https://www.cancer.org/cancer/types/thyroid-cancer/causes-risks-prevention/risk-factors.html>
